# Deletion of the WD40 Domain of LRRK2 in Zebrafish Causes Parkinsonism-Like Loss of Neurons and Locomotive Defect

**DOI:** 10.1371/journal.pgen.1000914

**Published:** 2010-04-22

**Authors:** Donglai Sheng, Dianbo Qu, Ken Hon Hung Kwok, Seok Shin Ng, Adrian Yin Ming Lim, Sharon Siqi Aw, Charlie Wah Heng Lee, Wing Kin Sung, Eng King Tan, Thomas Lufkin, Suresh Jesuthasan, Mathavan Sinnakaruppan, Jianjun Liu

**Affiliations:** 1 Department of Human Genetics, Genome Institute of Singapore, A*STAR, Singapore, Singapore; 2 Department of Stem Cell and Developmental Biology, Genome Institute of Singapore, A*STAR, Singapore, Singapore; 3 Department of Computational and Mathematical Biology, Genome Institute of Singapore, A*STAR, Singapore, Singapore; 4 National Neuroscience Institute and Duke–NUS Graduate Medical School, Singapore, Singapore; 5 Neuroscience Research Partnership, A*STAR, Singapore, Singapore; 6 Department of Physiology, National University of Singapore, Singapore, Singapore; Duke University, United States of America

## Abstract

LRRK2 plays an important role in Parkinson's disease (PD), but its biological functions are largely unknown. Here, we cloned the homolog of human LRRK2, characterized its expression, and investigated its biological functions in zebrafish. The blockage of zebrafish LRRK2 (zLRRK2) protein by morpholinos caused embryonic lethality and severe developmental defects such as growth retardation and loss of neurons. In contrast, the deletion of the WD40 domain of zLRRK2 by morpholinos targeting splicing did not induce severe embryonic developmental defects; rather it caused Parkinsonism-like phenotypes, including loss of dopaminergic neurons in diencephalon and locomotion defects. These neurodegenerative and locomotion defects could be rescued by over-expressing zLRRK2 or hLRRK2 mRNA. The administration of L-dopa could also rescue the locomotion defects, but not the neurodegeneration. Taken together, our results demonstrate that zLRRK2 is an ortholog of hLRRK2 and that the deletion of WD40 domain of zLRRK2 provides a disease model for PD.

## Author Summary

Parkinson's disease (PD) is a degenerative disease of the brain (central nervous system) that often impairs motor skills, speech, and other functions. PD was long thought to be caused by environmental factors, but the discovery of several gene mutations in the patients (mostly with familial form of PD) clearly demonstrated the involvement of genetic factors in the development of PD. Among the identified genes, LRRK2 was discovered to be one of the most important genetic causes of PD. The biological function of LRRK2 was, however, largely unknown. In this study, we studied the function of LRRK2 in zebrafish by blocking the normal function of LRRK2. The zebrafish showed features of neurodegeneration and locomotion defects, similar to those of PD patients. The defects of the fish could be rescued by expressing the normal protein of LRRK2, and the locomotion defect could also be rescued by the administration of L-dopa that is commonly used for treating PD patients. We have therefore developed a zebrafish model for PD that can be used for understanding the mechanism underlying the development of PD and will be helpful for future screening of new drugs to treat PD.

## Introduction

Parkinson's disease (PD) is a common neurodegenerative disorder characterized by the selective loss of dopaminergic neurons of the substantia nigra pars compacta (SNpc) and movement symptoms, including resting tremor, rigidity and postural instability [1]. The vast majority of PD patients are idiopathic, but a small number of patients show a familial inheritance where mutations in α-synuclein, Parkin, DJ-1, ubiquitin-C-hydrolase-L1 (UCHL1) or Leucine-rich repeat kinase 2 (LRRK2) play an important role [2], [3].

Of the identified disease genes for PD, mutations in LRRK2 are the most prevalent in both familial and sporadic PD patients [4]–[6], and show an interesting diversity in terms of population distribution as well as functional impact. For example, G2019S variant within the kinase domain was found to be a high-penetrate gain-of-function mutation (associated with enhanced kinase activity); it appears to be the most common mutation in the majority of the populations studied except Asian ones [7]–[9]. In contrast, G2385R variant within the WD40 domain results in a loss-of-function mutation (associated with the reduced kinase activity) and is a common susceptibility allele in Asian populations, but absent in Caucasians [10], [11]. The diverse spectrum of pathogenic mutations within the multiple domains of LRRK2 protein (see below) and the complex mechanisms by which these mutations influence the development of PD suggest that LRRK2 may be a master regulator of the disease development [12]. LRRK2 is therefore not only clinically important to link the familial and sporadic forms of PD, but also biologically significant for understanding the etiology of the disease [13].

The biological function of LRRK2 is, however, largely unknown. Human LRRK2 (hLRRK2) encodes an unusually large protein composed of multiple functional domains, including armadillo repeats, ankyrin repeats, two enzymatic S/T kinase and Roc GTPase domains, COR and WD40 domains (as the dimerization motif), indicating that LRRK2 is a complex multifunctional protein [14], [15]. The functional studies of LRRK2 were largely carried out by over-expressing either the wild-type or mutant allele of the MAPKKK and ROC domains of hLRRK2 in *in vitro* and *in vivo* model systems [16]. These transgenic studies suggested that the hyperactive kinase activity of LRRK2 may be cytotoxic and cause neurodegeneration. However, over-expression of the wild-type human LRRK2 protein does not always achieve the same mutant effect, as demonstrated in rat studies [17], raising a question on how much can be inferred from such ‘gain-of-function’ analysis of mutant alleles in understanding the normal function of LRRK2. When measuring the kinase activity of LRRK2 mutations using moesin as substrate [18], the most frequent mutation, G2019S, is the only one showing stimulated kinase activity. Hence, the mechanism by which LRRK2 mutation induces PD is more complex than previously imagined and is not only due to an increase in LRRK2 kinase activity. Analysis of loss-of-function mutations in *Drosophila* (dLRRK2) revealed conflicting findings; and it was not clear whether the disruption of dLRRK's function caused parkinsonism-like neurodegeneration and locomotive defects in this model system [19], [20]. So far, there are no reports on the function of WD40 or other domains in vertebrate models, such as the mouse or rat and the decreased kinase model for LRRK2 is very limited.

In this study, we performed the first *in vivo* loss-of-function study of LRRK2 in zebrafish. We cloned the zebrafish homolog of human LRRK2 and performed a series of molecular and genetic analyses to characterize its expression and biological functions, particularly the role of the WD40 domain, in embryonic and neuronal development.

## Results

### Molecular cloning of the zebrafish ortholog of hLRRK2

**Figure 1:**
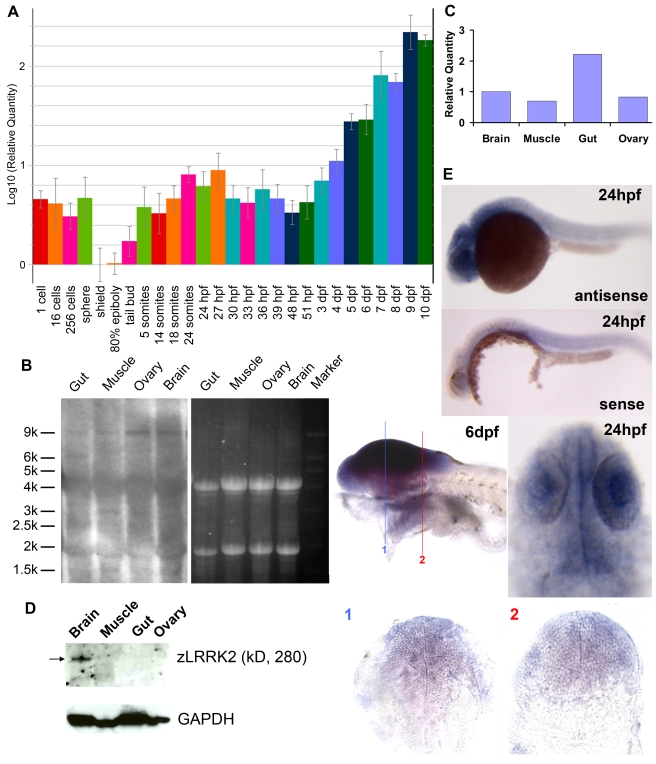
Expression profiling of *lrrk2* in zebrafish. (A) Quantitative RT–PCR analysis of *zlrrk2* mRNA expression from 1 cell stage to 10 dpf. (B–D) Northern blot analysis (B), quantitative RT–PCR (C), and Western blot analyses (D) of zLRRK2 expression in gut, muscle, ovary, and brain of adult fish. (E) In situ hybridization of *zlrrk2* mRNA at 24 hpf and 6 dpf stage. For 6 dpf stage, cryo-sectioning was performed in the position labeled as 1 and 2.

**Figure 2:**
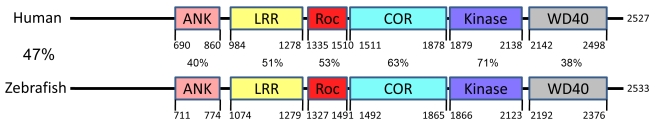
Conservation of amino acid sequences and functional domains of LRRK2 between zebrafish and human.

Through a TBLASTN analysis of hLRRK2 protein sequence against zebrafish cDNA sequences and a subsequent TBLASTX analysis of the identified zebrafish cDNA sequences against human cDNA sequences, we identified XM_682700 as the zebrafish homolog of hLRRK2. To clone the full length transcript of zLRRK2, we performed further RACE analysis using mRNAs isolated from the brain of adult fish and identified a 9168 bp transcript carrying both start and stop codons. The size of this transcript matched the *zlrrk2* mRNA detected by Northern analysis (Figure 1B). This 9168 bp transcript consists of 51 exons, spanning 118 kb genomic sequences (chr25:37299901–37361611, UCSC Genome Browser, Dec 2008) and encodes a protein of 2533 amino acid residues (Text S1). This experimentally cloned full length transcript is different from the ensembl predicted cDNA of 7410 bp that consists of 59 exons and encodes a protein with 2470 amino acid residues. The zLRRK2 protein contains all the functional domains of the hLRRK2 protein. There is a high degree conservation of amino acid sequences between the zLRRK2 and hLRRK2 proteins, with the highest conservation within the kinase domain (71%) (Figure 2). A phylogenetic analysis (Figure S1) revealed that the zLRRK2 protein was clustered together with the hLRRK2 protein as well as the LRRK2 proteins of other animal species.

### Analysis of expression profile of zLRRK2 in zebrafish

A temporal expression analysis by quantitative RT-PCR (qRT-PCR) (Figure 1A) indicated that the maternal mRNA of *zlrrk2* could be detected at the pre-MBT (mid blastula transition: from one cell to sphere) stages and was then degraded by the beginning of the gastrula stage. The zygotic expression of *zlrrk2* was first detectable at the tail bud stage (the last stage of gastrulation) and increased gradually during the segmentation and pharyngula stages, reaching a peak around 24 hours post fertilization (hpf). After a short period of reduction, the expression of *zlrrk2* increased again through the hatching and larval stages up to, at least, 10 days post fertilization (dpf). At both 24 hpf and 6 dpf, a strong expression of *zlrrk2* could be detected in the brain by whole mount in situ hybridization (WISH) analysis, and *zlrrk2*'s expression in the brain is ubiquitous (Figure 1E). In adult fish (older than three months), *zlrrk2* mRNA was detected in the brain, muscle, ovary and gut by Northern blot and qRT-PCR analyses (Figure 1B and 1C), but full-length zLRRK2 protein was predominantly detected in brain by Western blot (Figure 1D).

### Knockdown of zLRRK2 protein expression by morpholinos caused a severe embryonic defect and a loss of diencephalon tyrosine hydroxylase-positive (TH+) neurons

Microinjection of morpholinos targeting the ATG start site into embryos effectively abolished the expression of zLRRK2 protein, as determined by Western blot analysis (Figure S2 and Figure S3A). Knockdown of zLRRK2 expression resulted in severe embryonic lethality (∼90% of 64 embryos examined) within 3 dpf. The surviving morphants showed developmental retardation, such as slow growth, reduced brain size and heart edema compared to the wild type fish (Figure S3). WISH analysis showed a loss of TH+ neurons in the diencephalon of the surviving morphants (Figure S3B), which was consistent with the reduced level of tyrosine hydroxylase detected by the Western blot analysis (Figure S3A). Both heart edema and TH+ neuron loss phenotypes are morpholino concentration-dependent and can be partially rescued by over-expression of human LRRK2 (Figure S3 and Figure S4) (zLRRK2 was not used for the rescue, because its expression will be blocked by ATG morpholinos). However, due to the developmental retardation, it is not clear whether the loss of TH+ neurons in the diencephalon is an indication for a specific role of zLRRK2. The severe embryonic defect of the zLRRK2 knockdown also prevented us from studying its impact on locomotive movement.

### WD40 domain deletion caused neurodegeneration, including the loss of dopaminergic (DA) neurons and axon tract disorganization in brain

**Figure 3:**
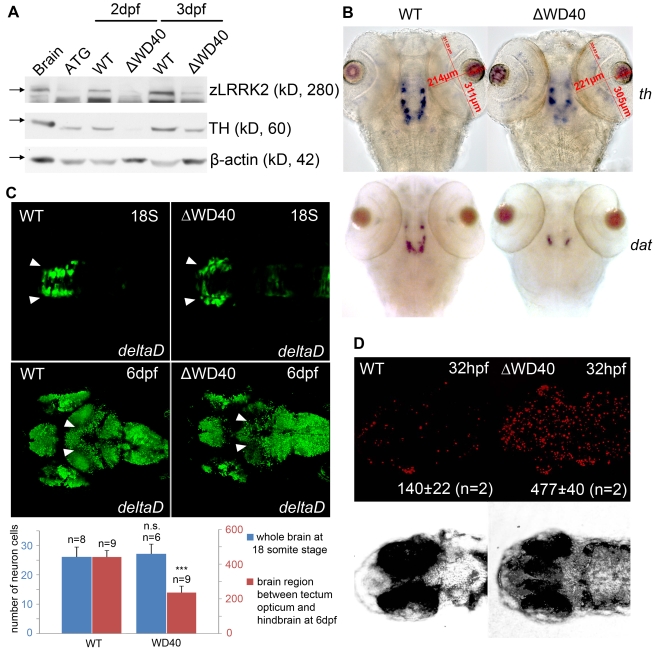
Phenotype of neuronal loss in LRRK2 ΔWD40 morphants. (A) Western blot analysis at 2 dpf and 3 dpf confirming the knockdown effect of zLRRK2 wild-type protein expression as well as the decreased TH protein expression in WD40 deletion morphant. Adult brain protein was used as positive control, and the ATG morphant at 3 dpf was used as negative control. The quantified results are shown in Figure S2. (B) WISH analysis at 3 dpf showing that WD40 domain deletion caused decreased TH and DAT expressions in WD40 deletion morphant, but without significant brain developmental retardation. (C) Uninjected (left) and WD40 deleted zebrafish expressing Kaede under the deltaD promoter. While neuron numbers is not much changed at 18 somite stage, there are fewer neurons in the midbrain of these 6 dpf fish, as indicated by the arrowheads. ***P<0.001, n.s.: not significant (unpaired Student's *t*-test). (D) Cell apoptosis assay of uninjected (left) and WD40 delete zebrafish embryos.

It has been shown that G2385R variant within the WD40 domain was associated with a very moderate risk for PD development [21]. We therefore hypothesize that the deletion of WD40 domain may lead to a weaker phenotype than the translational block of zLRRK2 expression, allowing us to study the specific role of zLRRK2 in neurodevelopment and locomotive movement. To delete the WD40 domain, we designed morpholinos that specifically interrupted the splicing of the 45th exon of *zlrrk2* and consequently introduced a pre-mature stop codon just upstream of the WD40 domain (Figure S5A). Delivery of this splicing-blocking morpholinos into embryos caused a production of truncated *zlrrk2* mRNA without the WD40 domain (zLRRK2-ΔWD40), as confirmed by RT-PCR and sequencing analyses (Figure S5B and S5C). As hypothesized, zLRRK2-ΔWD40 morphants showed a largely normal embryonic development, at least up to 7 dpf, without any distinguishable morphological defects, except a mild blood accumulation between the swimbladder and yolksac (Figure S6). Western blot analysis of whole fish lysate of zLRRK2-ΔWD40 morphants (3 dpf) (Figure 3A and Figure S2) showed significant loss of full-length (280 KD) zLRRK2 protein and TH protein expression. The reduction of TH expression was also confirmed by qRT-PCR analysis (Figure S5D). Consistently, the WISH analysis (at 3 dpf) showed a loss of TH+/DAT+ DA neurons in the diencephalon of the zLRRK2-ΔWD40 morphants (Figure 3B). As expected, the phenotypes of the zLRRK2-ΔWD40 morphants are morpholino concentration-dependent (Figure S4).

**Figure 4:**
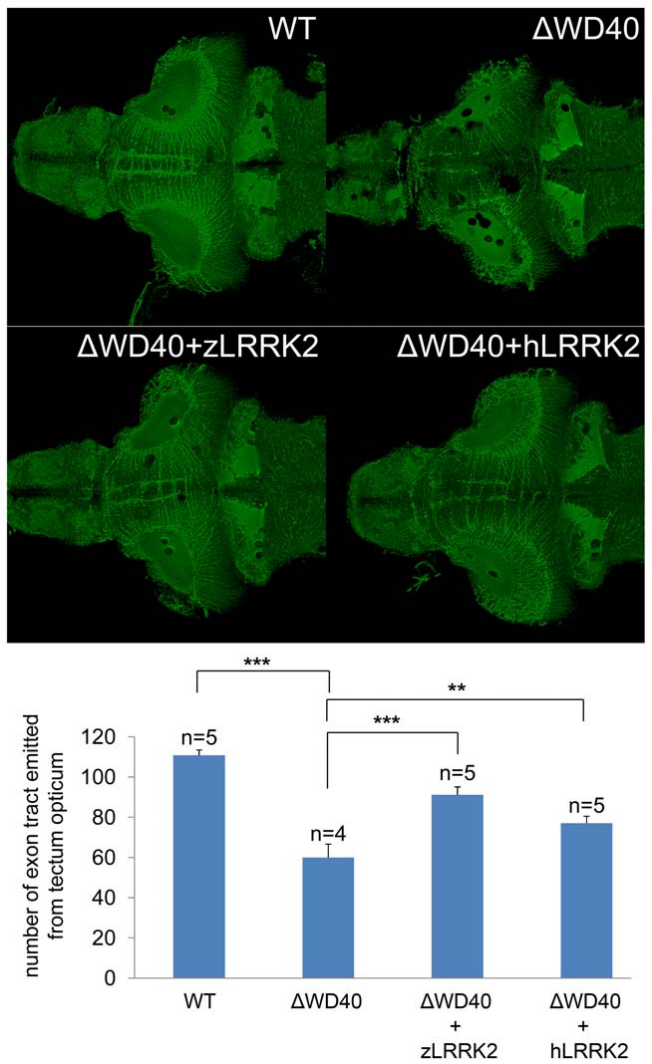
Analysis by acetylated tubulin staining of embryos at 6 dpf. Compared to the uninjected embryo, the WD40 deletion morphant has reduced and disorganized axon tracts in the midbrain of zebrafish. This phenotype could be rescued by over-expressing either zLRRK2 (WD40+zLRRK2) or hLRRK2 (WD40+hLRRK2) mRNA. **P<0.01, ***P<0.001 (unpaired Student's *t*-test).

To further investigate the impact of zLRRK2-ΔWD40 on neurodevelopment, we microinjected the zLRRK2-ΔWD40 morpholinos into the embryos of the Tg(DeltaD∶GAL4/UAS∶Kaede) line [22], where neurons are labeled by Kaede expression (driven by detlaD promoter). At 18 somite stage of embryonic development, no obvious neuron cell loss could be observed in the ΔWD40 morphants (compared to the wild-type fish) (Figure 3C). At 6 dpf, the forebrain and hindbrain of the morphants appeared to be normal and indistinguishable from the control siblings, but the midbrain, particularly the optic tectum of the morphants contained far fewer neurons than the control siblings (Figure 3C). Using the TUNEL assay, we found an enhanced apoptosis throughout the zLRRK2-ΔWD40 morphants (Figure 3D). We also stained axonal microtubules using an acetylated-tubulin antibody and found a reduction and disorganization of axon tracts, most prominently in the optic tectum of the zLRRK2-ΔWD40 morphants (Figure 4). These results indicated that the deletion of the WD40 domain causes the loss of neurons and the reduction and disorganization of axon tracts in the brain, including the DA loss in the diencephalon of the morphants.

**Figure 5:**
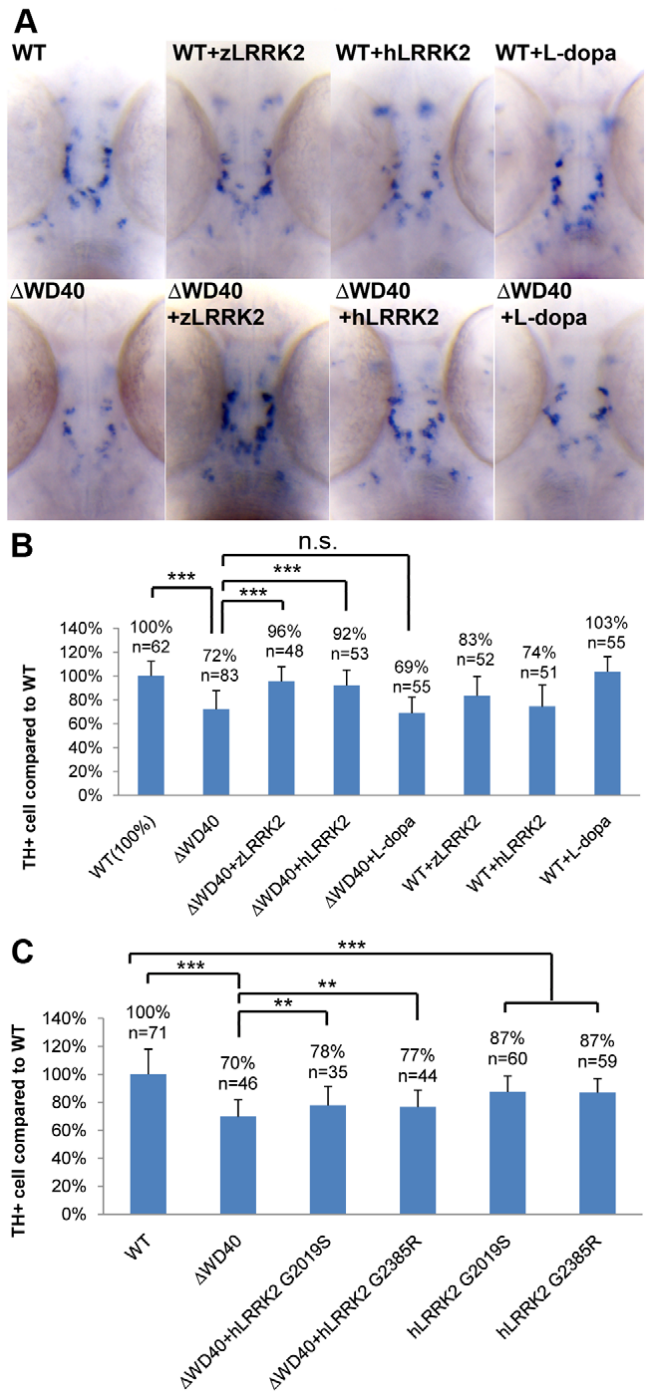
WISH Analysis of the loss of TH+ cell in WD40 morphant and its rescue by over-expressing wild-type zLRRK2 or hLRRK2 as well as hLRRK2 mutant alleles. (A) WISH analysis at 3 dpf shows the decreased TH expression in WD40 deletion morphant as well as the successful rescue by over-expressing either zLRRK2 or hLRRK2. The analysis also shows the decreased TH expression in the fish with over-expression of wild-type zLRRK2 and hLRRK2. This neurodegenerative phenotype could not be rescued by the treatment of L-dopa. (B) Quantification of rescue effect of zLRRK2 or hLRRK2 on TH+ cell loss in WD40 morphant. ***P<0.001 (unpaired Student's *t*-test). (C) Quantification of rescue effect of hG2019S or hG2385R on TH+ cell loss in WD40 morphant. **P<0.01, ***P<0.001 (unpaired Student's *t*-test).

Over-expression of either wild-type zLRRK2 or hLRRK2 (Figure S7) could rescue both the DA neuron loss (Figure 3B, Figure 5A and 5B) and axon tract disorganization (Figure 4) of the zLRRK2-ΔWD40 morphants, confirming that the neurodegenerative phenotypes of the zLRRK2-ΔWD40 morphant was a specific effect of the WD40 domain deletion due to splicing-blocking morpholinos, instead of off-target effect or unspecific toxicity of morpholinos. Furthermore, the successful rescue of the neurodegenerative phenotype by wild-type hLRRK2 confirmed zLRRK2 to be the functional ortholog of hLRRK2.

### The WD40 domain deletion of zLRRK2 caused a locomotion defect

**Figure 6:**
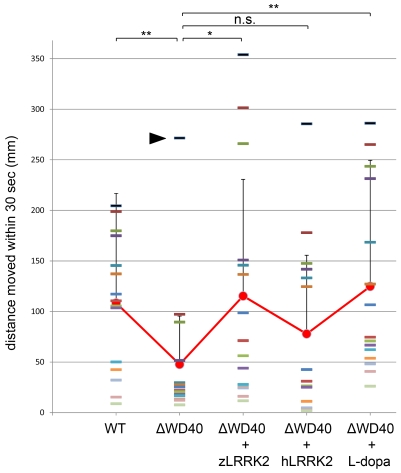
Analysis of locomotive movement by measuring the swimming distance in 30 seconds. The WD40 morphants swam much less distance than the wild-type fish. This locomotive defect could be rescued by over-expressing either zLRRK2 (WD40+zLRRK2) or hLRRK2 (WD40+hLRRK2) mRNA as well as by L-dopa treatment (WD40 + L-dopa). Each bar indicates the distance of an individual fish moved within 30 seconds. Each red dot (connected by red line) indicates the average distance of each of the five fish pools moved within 30 seconds, and the bar represents the SD of distance. *P<0.05, **P<0.01, n.s.: not significant (unpaired Student's *t*-test). It is noticeable that one of the ΔWD40 fish showed a very different phenotype from the rest. The distinct phenotype of this ‘outlier’ fish was likely due to either the failure or in-efficient knock-down effect by morpholino. The rescue effect by over-expressing hLRRK2 became statistically significant (P = 0.03) when the possible outlier of the ΔWD40 group (black arrow) is removed from the statistical analysis.

To investigate the locomotion behavior of the zLRRK2-ΔWD40 morphants, we measured the swimming distance of larval fish within time windows of 30 seconds. As shown in Figure 6, the zLRRK2-ΔWD40 morphants moved much smaller distances than the wild-type fish. Like the neurodegenerative defects, this reduced swimming activity could be rescued by over-expressing either zLRRK2 or hLRRK2 (Figure 6 and Figure S8). Intriguingly, this reduced swimming activity of zLRRK2-ΔWD40 morphants could also be rescued by the administration of Levo-dopa (L-dopa), a compound that is widely used to treat PD (Figure 6 and Figure S8). The administration of L-dopa, however, did not rescue the neurodegenerative phenotype of the morphants, as demonstrated by TH labeling (Figure 3B, Figure 5A and 5B).

### Over-expression of human LRRK2 G2019S and G2385R mutant alleles in zebrafish

In addition to the investigation of the WD40 deletion, we also investigated the impact of the over-expression of human LRRK2 G2019S and G2385R mutant alleles in zebrafish. The over-expression of both the mutant alleles could induce a similar blood accumulation between the swim bladder and yolk sac as the zLRRK2-ΔWD40 deletion and a mild loss of TH+ cell compared to wild-type (Figure S4 and Figure S9). Furthermore, unlike the wild-type zLRRK2 and hLRRK2, both hG2019S and hG2385R alleles could only partially rescue the loss of TH+ neurons in the zLRRK2-ΔWD40 morphants (Figure 5C).

## Discussion

In this study, we provide strong evidence that zLRRK2 is an ortholog of hLRRK2. The proteins of zLRRK2 and hLRRK2 show a conservation of amino acid sequence and share an identical domain structure. Phylogenetically, zLRRK2 is clustered together with hLRRK2, instead of hLRRK1, as well as the LRRK2 proteins of other animal species. Finally, the successful rescue of the defects of zLRRK2 morphants, in terms of both neurodegeneration and swimming abnormality, by over-expressing hLRRK2 mRNA, provides the most convincing evidence for a functional conservation of LRRK2 between zebrafish and human.

zLRRK2 shows a dynamic expression profile in zebrafish. During embryonic development, zLRRK2 transcript is mainly restricted to the brain, but demonstrating ubiquitous expression within brain, as observed in mouse, rat and human brains [23], [24]. In adult fish, *zlrrk2* mRNA was expressed in multiple tissues or organs. Western blot analysis (using an antibody against the WD40 domain), however, showed a rather restricted expression of zLRRK2 protein in the brain. Together with the previous finding that the LRRK2 protein isolated from transgenic mouse brain showed a higher kinase activity than from transgenic mouse lung or transfected cultured cells [25] and the suggestion that hLRRK2 has several splicing forms (AceView [26]); the differential expression patterns of zLRRK2 mRNA and protein in various tissues may suggest a complex mechanism for regulating zLRRK2 splicing and expression.

zLRRK2 plays an important role in neuronal development. The involvement of zLRRK2 in neurodevelopment is first suggested by the retarded brain development and the loss of TH+ neurons in the zLRRK2 ATG morphants and further evidenced by the neurodegenerative phenotypes of the zLRRK2-ΔWD40 deletion. The zLRRK2-ΔWD40 deletion caused a significant loss of DA neurons in the diencephalon, and other types of neurons are also likely affected. Interestingly, our preliminary study showed that the zLRRK2-ΔWD40 deletion had a rather limited impact on the development of neurons during early embryonic development. Considering that, 1) zLRRK2 shows a ubiquitous expression in the brain, 2) the zLRRK2-ΔWD40 deletion leads to an increased apoptosis activity across the brain, and 3) the midbrain, particularly the optic tectum of zebrafish is very stress-sensitive, the loss of neurons in the zLRRK2-ΔWD40 morphans is more likely due to a neurodegeneration process instead of the interruption of normal neuronal development. The loss of DA neurons in zLRRK2-ΔWD40 deletion likely happens as a result of a rather broad neurodegeneration within several regions of the brain. We speculate that LRRK2 may be important for neuron survival and thus play a more prominent role in neural maintenance, rather than development of neurons. The interruption of normal LRRK2 function may cause neurons to become more sensitive to factors that might trigger cell death.

The zLRRK2-ΔWD40 deletion also caused a significant reduction and disorganization of axon tracts, more prominently in the midbrain. This is consistent with the previous finding from transgenic mouse study that LRRK2 is involved in neurite growth [27]. Since LRRK2 interacts with microtubule through Roc domain [12] and the WD40 domain can bind to the Roc domain [28], the reduced and disorganized axon tracts in the midbrain of the zLRRK2-ΔWD40 morphants may be due to an interruption of the microtubule cytoskeleton [29]. This would be consistent with the well established requirement for microtubules in axon outgrowth. Our result has also supported the recent hypothesis that Parkinsonism may be due to a disorganized ‘microtubule railroad’ system, which can be the consequence of a faulty of motor [30] or perhaps microtubule. However, further study will be needed to elucidate whether the reduced and disorganized axon tracts truly reflects the interrupted ‘microtubule railroad’ system and thus causes neurodegeneration.

This is the first demonstration of the role of the WD40 domain of LRRK2 in neural development and/or neural maintenance. The WD40 domain is known to mediate protein-protein interaction in many contexts, such as signal transduction, transcription regulation, cell cycle control, apoptosis and cytoskeleton assembly [31]. The WD40 domain has been suggested to play a crucial role in LRRK2 self-interaction and autophosphorylation, which regulates the kinase activity of LRRK2 [28]. The deletion of the WD40 domain causes a partial reduction in kinase activity *in vitro*, which could be restored to a normal level by the over-expression of the gain-of-function mutation R1441C [32]. We have previously shown that the G2385R risk variant in the WD40 domain increases neuronal apoptosis under cellular stress [33], providing further support for the functional role of WD40 domain. However, the different phenotypic impact of blocking the kinase activity of LRRK2 (by knocking-down the protein expression) and deleting the WD40 domain suggests that LRRK2 may influence the neurodevelopment through other mechanisms beyond the modification of its kinase activity.

We have demonstrated a locomotion defect in the zLRRK2-ΔWD40 morphant. More importantly, we confirmed that the locomotion defect likely happens as a direct result of the dopamine insufficiency (due to the loss of DA neurons), since the defect can be rescued by supplementing dopamine through the administration of L-dopa. The administration of L-dopa did not rescue the loss of DA neurons in the diencephalon of zLRRK2-ΔWD40 morphant. This is consistent with the therapeutic effect of L-dopa in treating human PD condition where the treatment can only offer a temporary relieve of clinical symptoms, but cannot restore the degeneration of DA neurons [34]. The morphant phenotypes of the zLRRK2-ΔWD40 deletion seem to closely mimic the human condition of PD at both molecular and physiological levels. In addition, our preliminary study has shown that the over-expression of human point mutations, such as G2019S and G2385R, shows a similar impact on neural development as the WD40 deletion in zebrafish. This is consistent with the dominant effect of human point mutations, such as G2019S, to induce PD-like phenotypes in other animal models. Furthermore, MPTP treatment was shown previously to trigger the similar degeneration of DA neurons and locomotion behavior defects in zebrafish as in human [35], [36]. Taken together, these studies have demonstrated that zebrafish can be used for studying PD-related neurodegeneration, and the deletion of WD40 domain in zebrafish provides a potential disease model for PD.

It is noteworthy to point out that the neuron loss of zLRRK2-ΔWD40 can be observed as early as in the late stage of embryonic development. As a limitation of this model, the early-onset phenotype of zLRRK2-ΔWD40 does not fully recapitulate the late-onset PD phenotype in human. This difference may, at least partially, due to the fact that the knock-down effect of splice-blocking morpholino could be up to 90% in zebrafish, whereas PD patients usually carry heterozygous point mutations of LRRK2. Consequently, the early-onset phenotype of zLRRK2-ΔWD40 may be due to more severe mutational effect of WD40 deletion than heterozygous point mutations in human. Furthermore, although the molecular function of LRRK2 is conserved between zebrafish and human, the neuronal system, including dopaminergic one, may not be fully conserved between two species. As a consequence, mutations of functionally conserved LRRK2 may show partially different phenotypes. It is not truly unexpected because it is rather uncommon for animal models to recapitulate the full phenotype of human disease.

Several animal models for PD were developed in recent years. DJ-1 knockout mice show decreased motor functions, increased striatal dopamine level without the loss of DA neurons [37], [38] and increased sensitivity to MPTP and oxidative stress [39]. Consistently, the knockdown of DJ-1 expression in zebrafish did not result in a loss of DA neurons, unless under the exposure to pro-oxidant hydrogen peroxide and the proteasome inhibitor MG132 [40]. In PINK1 knockdown [41] or knockout mice [42], there were no changes in striatal dopamine level, nigral DA neurons numbers and motor activity. In Parkin knockout mice, mutants with the deletion of exon2 [42] showed no abnormalities compared to wild-type mice in terms of the nigral DA neurons numbers and motor activity, while the mutants with the deletion of exon3 [43] showed behavioral deficits, but without DA neurons loss. The conditional LRRK2 G2019S model [27] was reported to have no obvious neuropathological or motor abnormalities at 12 months of age. Over-expression of UCH-L1 in zebrafish did not result in a discernible phenotypic effect [44]. Therefore, the previous vertebrate models did not show, in a consistent fashion, the progressive loss of nigrostriatal dopaminergic neurons and motor defects. In *Drosophila*, the expression of wild-type and mutant forms of human α-synuclein lead to a progressive DA neuron loss [45], and the loss could be suppressed by the over-expression of parkin [46]. *Drosophila* parkin-null mutants also showed motor deficits [47] and DA neuron degeneration [48], [49]. Recently, Lee *et al.* found that the loss of Lrrk2 in *Drosophila* lead to impaired locomotive activity and degeneration of DA [20]. However, Wang *et al's* study did not confirm this observation and instead found an increased sensitivity to oxidative stress [19]. The over-expression of hLRRK2 wild-type or G2019S mutant allele in *Drosophila* resulted in loss of DA neurons, locomotor dysfunction and early mortality, which could be rescued by the administration of levodopa [50]. Although this invertebrate model recapitulates several features of human PD, a recent study showed that dLRRK2 is not an ortholog of hLRRK2 [51], dampening the relevance and importance of this *Dorsophila* LRRK2 model for PD.

In summary, we have demonstrated that zLRRK2 is an ortholog of hLRRK2. As a vertebrate model, the zLRRK2-ΔWD40 morphant recapitulates some key molecular, physiological and behavioral hall-marks of PD. Together with other animal models, this potential vertebrate model provides opportunities to investigate the biological mechanisms underlying the development of PD. The fact that the locomotion defect of the zLRRK2-ΔWD40 morphant can be ‘treated’ by L-dopa also raises the possibility that this zebrafish model may be used for screening new drugs to treat PD.

## Materials and Methods

### Molecular cloning of full-length zebrafish LRRK2 cDNA

A TBLASTN analysis of the human LRRK2 protein against zebrafish cDNA sequences yielded two hits, XM_682700 and XM_682192. Through reciprocal TBLASTX, we found that XM_682700 was the possible homolog of human LRRK2 (hLRRK2), whereas XM_682192 was the possible homolog of human LRRK1. As indicated, XM_682700 is a predicted cDNA of zebrafish LRRK2 (zLRRK2) with about 6 kb sequences. To verify whether it is a true coding gene, we blasted this sequence against the genome of zebrafish and identified 6 EST sequences (BI884532, EB935015, BI882500, AL918398, BQ258400 and CD758533). To identify the full length transcript of zLRRK2, we performed RACE analysis by using mRNA isolated from the brain of adult fish and the sequence of BI884532 (most 5′ end) for designing the primer of 5′-RACE and AL918398 (most 3′ end) for designing the primer of 3′-RACE. Likewise, using the sequences of the same two ESTs, a pair of primers was designed to amplify the middle part of the zLRRK2 transcript. After cloning and sequencing, we identified a 9168 bp transcript carrying both start and stop codons.

5′ RACE and 3′ RACE were performed by using the GeneRacer Kit (Invitrogen, USA) according to manufacturer instructions. Gene Specific Primer for 5′ end is 5′ CTGCATTTCAGCAACACAGG 3′ and Gene Specific Primer for 3′ end is 5′ AAGTCCAGCGTGTAGCTGAGCGTGGAAATG 3′.

### Quantitative RT–PCR

qRT-PCR was performed with HIGH CAPACITY CDNA REVERSE TRANSCRIPTION KIT and SYBR Green 1 PCR Master Mix. Gene specific primers are 5′GACTCCGAGGCGATACAG 3′ and 5′ CAAGGGCACTCAGACAGG 3′. Internal control beta-actin primers are 5′ TGGCAAAGGGAGGTAGTTG 3′ and 5′GTGAGGAGGGCAAAGTGG 3′.

### Zebrafish (*Danio rerio*) maintenance

Wild type AB line and Tg(DeltaD∶GAL4/UAS∶Kaede) line zebrafish were maintained according to methods described in The Zebrafish Book (Westerfield, 1995). Details are provided in Protocol S1.

### Whole-mount in-situ hybridization

Procedure was followed by the method described in The Zebrafish Book (Westerfield, 1995). Gene specific primers are: 5′ TGCAAACGGAGGTAAAAACC 3′ and 5′AGATGATCCTGGTCCCACAG 3′ for *zlrrk2*; 5′AAGGATGGCTTGGAGGAC3′ and 5′CTCGGAGGGTGGAGTAGA3′ for *th*. PCR product was cloned into pGEMT vector for probe synthesis. For *dat*, 5′GGGGTTCAGTTCACCTCCTC3′ and 5′CATTAACCCTCACTAAAGGGAAGACTCCATCCCTCCCATAGC3′ (with T3 promoter) were used for PCR and probe synthesis.

### Morpholinos

Three different morpholino antisense oligonucleotides (translation start site of *zlrrk2*, ATG–ACAACTCCTCTATTTCTGCCATGAT; intron 45 splice donor junction; EI–CACAAGCAGATTTATTAACCTGTGC; intron 44 splice acceptor junction, IE–GCTCCTGAAACACAGCATTAGGAAC) were obtained from Gene Tools (Philomath, OR) and injected at the one- to two-cell stage. Details of splicing interfering mopholino design are provided in Protocol S1. Efficacy of morpholinos directed against splice sites was evaluated using RT-PCR (Figure S5) with forward primers F1-5′TGCAAACGGAGGTAAAAACC 3′ and in conjunction with reverse primer R1- 5′AGATGATCCTGGTCCCACAG 3′. Dosage-dependent effects of morpholinos were observed (Figure S4).

### Northern analysis

Gene specific primers for probe synthesis are 5′GTTGGCGTTCTGCCGGGT CC 3′ and 5′ AAAGCGGCCGCATTAAGCAGCGTTTCTCTCATTCTGCGG 3′. Details are provided in Protocol S1.

### Western analysis

The anti-zLRRK2 antibody used in this study is developed from the C-terminal (within WD40 domain, CSTRKPKVHSEDQSR) regions of LRRK2. Western analysis was conducted using standard techniques. Details are provided in Protocol S1 and Figure S10. The truncated protein of zLRRK2-ΔWD40 cannot be recognized by this antibody due to its absence of the WD40 domain.

### Rescue experiments

For plasmid rescue, pCI-neo vector (Promega, USA) harboring *zlrrk2* or *hLRRK2* cDNAs tagged by Flag and *hLRRK2* G2019S or G2385R cDNAs tagged by myc were used. At 2 to 3 days after the microinjection of the linearized plasmids by SfiΙ, total fish homogenate was subjected to anti-Flag Western blot analysis, clearly showing that both the *zlrrk2* and *hLRRK2* cDNAs could be expressed in zebrafish (Figure S7). Upon confirming the expression of *zlrrk2* and *hLRRK2* cDNAs in zebrafish, the rescue experiment was performed by the co-microinjection of the plasmids and the ATG or splicing-blocking morpholinos into embryos. Dosage-dependent effects of plasmid over-expression were observed (Figure S4). For the rescue of the ATG morphants, only the *hLRRK2* cDNAs was used, because the ATG morpholinos will block the protein expression of the *zlrrk2* cDNA.

For L-dopa rescue, L-dopa (1 mM) (Sigma) was applied at 5 dpf stage and behavior test was performed at 6 dpf.

### Apoptosis assay

Apoptosis assay was carried out by using In Situ Cell Death Detection Kit, TMR red (Roche).

### Acetylated-tubulin staining on the brain

Procedures are referenced from Michael Hendricks and Suresh Jesuthasan's work [52]. Details are provided in Protocol S1.

### Imaging and analysis

On a Zeiss LSM510 META confocal microscope, live embryos' brains were imaged using an Achroplan 10X/0.30 water immersion objective and alpha tubulin stained brains were imaged using a EC Plan-Neofluar 10x/0.30 objective. Projection of confocal z-stacks was done using Zeiss software.

Video recording for the behavior analysis of 6 dpf larva were taken with a Sony HDR-SR12E. Behavior analysis was done using NIH ImageJ.

### Sequence conservation analysis

Protein domains of zebrafish LRRK2 were predicted by SMART (Simple Modular Architecture Research Tool, http://smart.embl-heidelberg.de). Each domains and whole protein of zLRRK2 and hLRRK2 were aligned by ClustalW2 (http://www.ebi.ac.uk/Tools/clustalw2/index.html).

### Phylogenetic analysis

The animal protein sequences of the COR and Kinase domains are obtained from Marin's study [14]. Details are provided in Protocol S1.

## Supporting Information

Figure S1 Phylogenetic analysis of LRRK2 by using either COR or kinase domain sequences.
(0.33 MB TIF)

Figure S2 Quantification analysis of the results in Figure 3A and Figure S3A.
(0.39 MB TIF)

Figure S3 Phenotypes of zLRRK2 ATG morphants. (A) Western blot analysis at 72 hpf showing the strong reduction in expression of zLRRK2 protein as well as the decreased TH protein level in embryos injected with ATG morpholino. The analysis was done in duplicate, and adult brain protein was used as positive control for zLRRK2. (B) WISH analysis at 2 dpf showing retarded brain development and decreased TH expression in an ATG morphant (right). An uninjected sibling is shown for comparison (left). Quantification of the TH+ cell loss in ATG morphant as well as the rescue of ATG morphant with hLRRK2 was shown on the right. Co-injection of hLRRK2 significantly rescue the number of TH+ cells compared to ATG MO alone. ***P<0.001 (unpaired Student's t-test). (C) Heart edema phenotype of ATG morphant and rescue effect of hLRRK2 on ATG morphant.
(4.98 MB TIF)

Figure S4 Dosage-dependent effect of morpholinos. (A) Dosage dependent experiment of WD40 morpholino and lrrk2 plasmid (zLRRK2, hLRRK2, hG2019S, and hG2385R) basing on the phenotype of blood accumulation. (B) Dosage dependent experiment of ATG morpholino basing on the quantification of TH+ cell loss. ***P<0.001 (unpaired Student's t-test).
(1.01 MB TIF)

Figure S5 RT-PCR Analysis to confirm the blockage of exon 45 splicing by WD40 morpholino. (A) Schematic representation of the exons 44 to 47 of zLRRK2 to show the morpholinos used for blocking the 45th exon splicing (the track above the exons) as well as the primers (the track below the exons) used in the RT-PCR verification of the splicing blockage effect at 2 dpf and 3 dpf. (B) RT-PCR analysis at 2 and 3 dpf showing that the WD40 morpholino could block the splicing of exon 45, and the major of transcripts were the abnormally spliced one without exon 45 (confirmed by sequence analysis). (C) The percentage of the normally spliced transcript in the total amount of normal and abnormally spliced (without exon 45) transcripts in WT and WD40 morphant at 2 and 3 dpf. The densities of normal and abnormal spliced transcripts (in B) at 2 and 3 dpf were measured, and the percentage of normally spliced transcript was calculated by comparing the density of normal transcript to the total density of both normal and abnormal transcripts (sum of density from normal and abnormal forms). The percentage is presented as mean ± SD from 4 independent experiments. In wild-type embryos, no abnormal splicing forms were detected. ***P<0.001 (unpaired Student's t-test). (D) Quantitative RT-PCR analysis of wild-type controls and WD40 morphants at 2 and 3 dpf, showing that both TH and WT zLRRK2 mRNA levels were reduced to 40%–60% of WT fishes.
(0.26 MB TIF)

Figure S6 Morphological phenotype of WD40 morphants (From 3 dpf to 7 dpf). Morphants show no significant morphological defects compared to WT fishes except the mild blood accumulation in gut and pronephric duct between the york sac and swimming bladder.
(0.91 MB TIF)

Figure S7 Linearized plasmid harboring either zLRRK2 or hLRRK2 cDNA tagged by Flag can be expression in zebrafish embryos by microinjection.
(0.04 MB TIF)

Figure S8 Swimming tracts of 15 fish from each of the five fish pools: wild-type (wt), WD40 morphants (WD40), WD40 morphants co-injected with plasmid harboring either zLRRK2 (WD40+zLRRK2) or hLRRK2 (WD40+hLRRK2) and WD40 morphants treated with levodopa (WD40+levodopa).
(0.81 MB TIF)

Figure S9 Morphological phenotype of hG2019S and hG2385R overexpression. At 6 dpf, hG2019S and hG2385R overexpression shows no significant morphological defects compared to WT except the blood accumulation in gut and pronephric duct between york sac and swimming bladder.
(0.60 MB TIF)

Figure S10 Analysis of zLRRK2 antibody specificity. (A) Specificity of zLRRK2 antibody specificity was verified by western blot analysis. Positive signals could be largely blocked by pre-incubating the anti-zLRRK2 antibody with the neutralizing peptide (+ peptide, left panel). Similarly, the zLRRK2 signal was completely blocked by pre-incubating the anti-zLRRK2 antibody with the neutralizing peptide in the zLRRK2-overexpressing Cos-7 cells (+ peptide, right panel). (B) Western blot analysis showing the specificity of the zLRRK2 antibody against zLRRK2 in Cos-7 cell line. Human LRRK2 and zLRRK2 recombinant proteins were overexpressed in Cos-7 cells separately and detected by anti-hLRRK2 (left panel) and anti-zLRRK2 (right panel), respectively. A positive band (280 kD, arrow) was only detected in hLRRK2-overexpressing cells (hLRRK2, left panel), using anti-hLRRK2 antibody (NOVUS NB 300–268). No obvious band was detected in zLRRK2-overexpressing and untransfected control cells (zLRRK2 and control, left panel); On the other hand, a sharp band (arrow) was detected in zLRRK2-overexpressing cells using the anti-zLRRK2 antibody (zLRRK2, right panel), but not in hLRRK2-overexpressing and untransfected control cells (hLRRK2 and control, right panel). This indicates the anti-zLRRK2 antibody is specifically against zLRRK2 proteins.
(0.11 MB TIF)

Text S1 cDNA sequence and exon information of zLRRK2.
(0.09 MB DOC)

Protocol S1 Supporting materials and methods.
(0.04 MB DOC)

## References

[1] Thomas B, Beal MF (2007). Parkinson's disease.. Hum Mol Genet.

[2] Douglas MR, Lewthwaite AJ, Nicholl DJ (2007). Genetics of Parkinson's disease and parkinsonism.. Expert Rev Neurother.

[3] Schapira AH (2006). Etiology of Parkinson's disease.. Neurology.

[4] Paisan-Ruiz C, Jain S, Evans EW, Gilks WP, Simon J (2004). Cloning of the gene containing mutations that cause PARK8-linked Parkinson's disease.. Neuron.

[5] Ross OA (2007). Lrrking in the background: common pathways of neurodegeneration.. J Am Geriatr Soc.

[6] Zimprich A, Biskup S, Leitner P, Lichtner P, Farrer M (2004). Mutations in LRRK2 cause autosomal-dominant parkinsonism with pleomorphic pathology.. Neuron.

[7] Fung HC, Chen CM, Hardy J, Hernandez D, Singleton A (2006). Lack of G2019S LRRK2 mutation in a cohort of Taiwanese with sporadic Parkinson's disease.. Mov Disord.

[8] Ozelius LJ, Senthil G, Saunders-Pullman R, Ohmann E, Deligtisch A (2006). LRRK2 G2019S as a cause of Parkinson's disease in Ashkenazi Jews.. N Engl J Med.

[9] Schapira AH (2006). The importance of LRRK2 mutations in Parkinson disease.. Arch Neurol.

[10] Di Fonzo A, Wu-Chou YH, Lu CS, van Doeselaar M, Simons EJ (2006). A common missense variant in the LRRK2 gene, Gly2385Arg, associated with Parkinson's disease risk in Taiwan.. Neurogenetics.

[11] Tan EK (2006). Identification of a common genetic risk variant (LRRK2 Gly2385Arg) in Parkinson's disease.. Ann Acad Med Singapore.

[12] Gandhi PN, Wang X, Zhu X, Chen SG, Wilson-Delfosse AL (2008). The Roc domain of leucine-rich repeat kinase 2 is sufficient for interaction with microtubules.. J Neurosci Res.

[13] Lesage S, Durr A, Brice A (2007). LRRK2: a link between familial and sporadic Parkinson's disease?. Pathol Biol (Paris).

[14] Marin I (2006). The Parkinson disease gene LRRK2: evolutionary and structural insights.. Mol Biol Evol.

[15] Mata IF, Wedemeyer WJ, Farrer MJ, Taylor JP, Gallo KA (2006). LRRK2 in Parkinson's disease: protein domains and functional insights.. Trends Neurosci.

[16] Gandhi PN, Chen SG, Wilson-Delfosse AL (2009). Leucine-rich repeat kinase 2 (LRRK2): a key player in the pathogenesis of Parkinson's disease.. J Neurosci Res.

[17] MacLeod D, Dowman J, Hammond R, Leete T, Inoue K (2006). The familial Parkinsonism gene LRRK2 regulates neurite process morphology.. Neuron.

[18] Jaleel M, Nichols RJ, Deak M, Campbell DG, Gillardon F (2007). LRRK2 phosphorylates moesin at threonine-558: characterization of how Parkinson's disease mutants affect kinase activity.. Biochem J.

[19] Wang D, Tang B, Zhao G, Pan Q, Xia K (2008). Dispensable role of Drosophila ortholog of LRRK2 kinase activity in survival of dopaminergic neurons.. Mol Neurodegener.

[20] Lee SB, Kim W, Lee S, Chung J (2007). Loss of LRRK2/PARK8 induces degeneration of dopaminergic neurons in Drosophila.. Biochem Biophys Res Commun.

[21] Tan EK (2007). The role of common genetic risk variants in Parkinson disease.. Clin Genet.

[22] Scheer N, Groth A, Hans S, Campos-Ortega JA (2001). An instructive function for Notch in promoting gliogenesis in the zebrafish retina.. Development.

[23] Higashi S, Biskup S, West AB, Trinkaus D, Dawson VL (2007). Localization of Parkinson's disease-associated LRRK2 in normal and pathological human brain.. Brain Res.

[24] Taymans JM, Van den Haute C, Baekelandt V (2006). Distribution of PINK1 and LRRK2 in rat and mouse brain.. J Neurochem.

[25] Li X, Tan YC, Poulose S, Olanow CW, Huang XY (2007). Leucine-rich repeat kinase 2 (LRRK2)/PARK8 possesses GTPase activity that is altered in familial Parkinson's disease R1441C/G mutants.. J Neurochem.

[26] Thierry-Mieg D, Thierry-Mieg J (2006). AceView: a comprehensive cDNA-supported gene and transcripts annotation.. Genome Biol.

[27] Wang L, Xie C, Greggio E, Parisiadou L, Shim H (2008). The chaperone activity of heat shock protein 90 is critical for maintaining the stability of leucine-rich repeat kinase 2.. J Neurosci.

[28] Greggio E, Zambrano I, Kaganovich A, Beilina A, Taymans JM (2008). The Parkinson disease-associated leucine-rich repeat kinase 2 (LRRK2) is a dimer that undergoes intramolecular autophosphorylation.. J Biol Chem.

[29] Melrose H (2008). Update on the functional biology of Lrrk2.. Future Neurol.

[30] Farrer MJ, Hulihan MM, Kachergus JM, Dachsel JC, Stoessl AJ (2009). DCTN1 mutations in Perry syndrome.. Nat Genet.

[31] Li D, Roberts R (2001). WD-repeat proteins: structure characteristics, biological function, and their involvement in human diseases.. Cell Mol Life Sci.

[32] Iaccarino C, Crosio C, Vitale C, Sanna G, Carri MT (2007). Apoptotic mechanisms in mutant LRRK2-mediated cell death.. Hum Mol Genet.

[33] Tan EK, Zhao Y, Skipper L, Tan MG, Di Fonzo A (2007). The LRRK2 Gly2385Arg variant is associated with Parkinson's disease: genetic and functional evidence.. Hum Genet.

[34] Nagatsua T, Sawadab M (2009). L-dopa therapy for Parkinson's disease: past, present, and future.. Parkinsonism Relat Disord.

[35] Bretaud S, Lee S, Guo S (2004). Sensitivity of zebrafish to environmental toxins implicated in Parkinson's disease.. Neurotoxicol Teratol.

[36] Lam CS, Korzh V, Strahle U (2005). Zebrafish embryos are susceptible to the dopaminergic neurotoxin MPTP.. Eur J Neurosci.

[37] Chen L, Cagniard B, Mathews T, Jones S, Koh HC (2005). Age-dependent motor deficits and dopaminergic dysfunction in DJ-1 null mice.. J Biol Chem.

[38] Goldberg MS, Pisani A, Haburcak M, Vortherms TA, Kitada T (2005). Nigrostriatal dopaminergic deficits and hypokinesia caused by inactivation of the familial Parkinsonism-linked gene DJ-1.. Neuron.

[39] Kim RH, Smith PD, Aleyasin H, Hayley S, Mount MP (2005). Hypersensitivity of DJ-1-deficient mice to 1-methyl-4-phenyl-1,2,3,6-tetrahydropyrindine (MPTP) and oxidative stress.. Proc Natl Acad Sci U S A.

[40] Bretaud S, Allen C, Ingham PW, Bandmann O (2007). p53-dependent neuronal cell death in a DJ-1-deficient zebrafish model of Parkinson's disease.. J Neurochem.

[41] Zhou H, Falkenburger BH, Schulz JB, Tieu K, Xu Z (2007). Silencing of the Pink1 gene expression by conditional RNAi does not induce dopaminergic neuron death in mice.. Int J Biol Sci.

[42] Kitada T, Pisani A, Porter DR, Yamaguchi H, Tscherter A (2007). Impaired dopamine release and synaptic plasticity in the striatum of PINK1-deficient mice.. Proc Natl Acad Sci U S A.

[43] Goldberg MS, Fleming SM, Palacino JJ, Cepeda C, Lam HA (2003). Parkin-deficient mice exhibit nigrostriatal deficits but not loss of dopaminergic neurons.. J Biol Chem.

[44] Son OL, Kim HT, Ji MH, Yoo KW, Rhee M (2003). Cloning and expression analysis of a Parkinson's disease gene, uch-L1, and its promoter in zebrafish.. Biochem Biophys Res Commun.

[45] Feany MB, Bender WW (2000). A Drosophila model of Parkinson's disease.. Nature.

[46] Haywood AF, Staveley BE (2004). Parkin counteracts symptoms in a Drosophila model of Parkinson's disease.. BMC Neurosci.

[47] Pesah Y, Pham T, Burgess H, Middlebrooks B, Verstreken P (2004). Drosophila parkin mutants have decreased mass and cell size and increased sensitivity to oxygen radical stress.. Development.

[48] Cha GH, Kim S, Park J, Lee E, Kim M (2005). Parkin negatively regulates JNK pathway in the dopaminergic neurons of Drosophila.. Proc Natl Acad Sci U S A.

[49] Whitworth AJ, Theodore DA, Greene JC, Benes H, Wes PD (2005). Increased glutathione S-transferase activity rescues dopaminergic neuron loss in a Drosophila model of Parkinson's disease.. Proc Natl Acad Sci U S A.

[50] Liu Z, Wang X, Yu Y, Li X, Wang T (2008). A Drosophila model for LRRK2-linked parkinsonism.. Proc Natl Acad Sci U S A.

[51] Marin I (2008). Ancient origin of the Parkinson disease gene LRRK2.. J Mol Evol.

[52] Hendricks M, Jesuthasan S (2007). Electroporation-based methods for in vivo, whole mount and primary culture analysis of zebrafish brain development.. Neural Dev.

