# Supplementary figures and images for: Deletion of the WD40 Domain of LRRK2 in Zebrafish Causes Parkinsonism-Like Loss of Neurons and Locomotive Defect
Source: PLoS Genet. 2010 Apr 22;6(4):e1000914. doi: 10.1371/journal.pgen.1000914 (PMC2858694; doi:10.1371/journal.pgen.1000914)

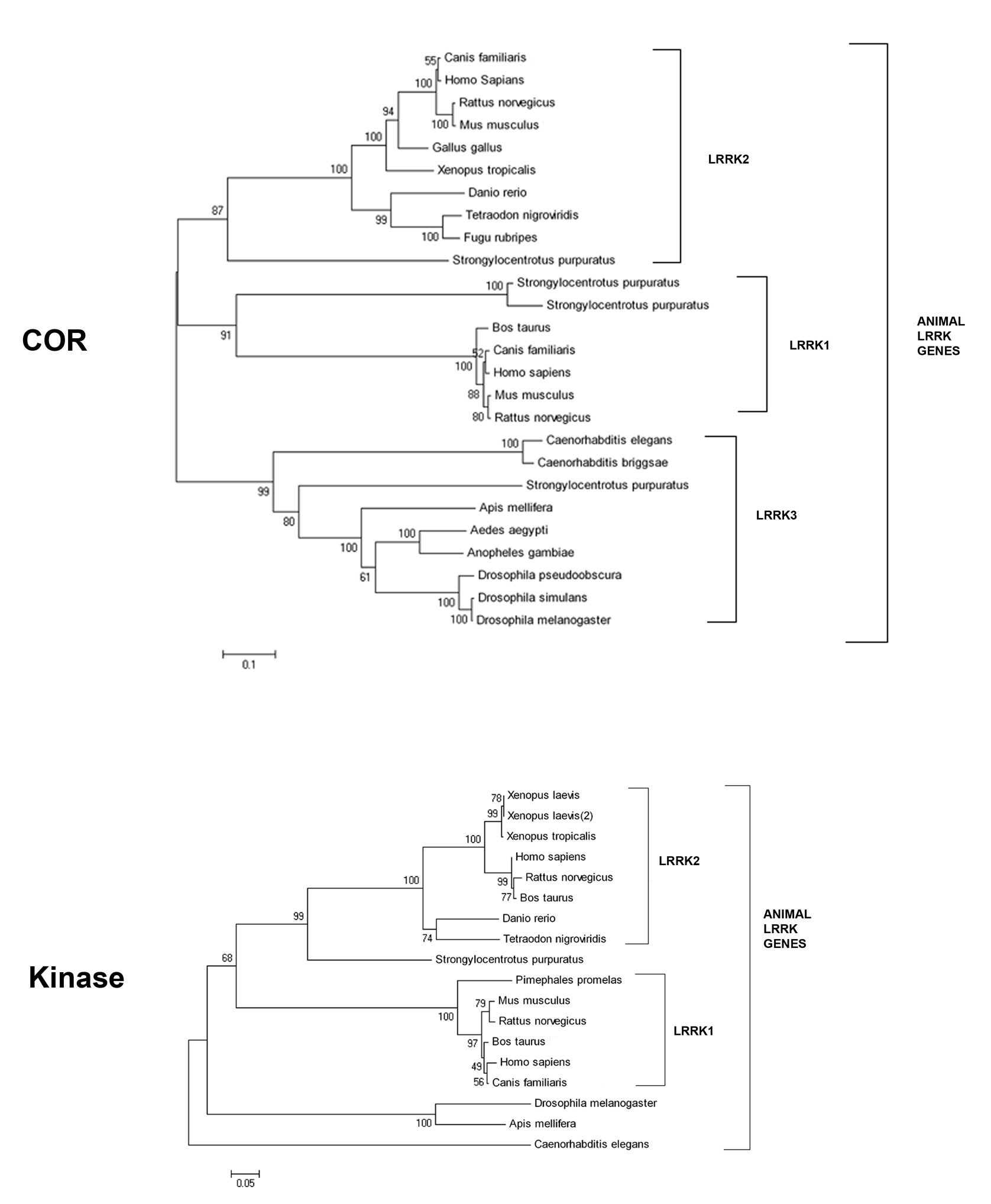

Supplement: Figure S1 — Phylogenetic analysis of LRRK2 by using either COR or kinase domain sequences. (0.33 MB TIF) [file pgen.1000914.s001.tif]

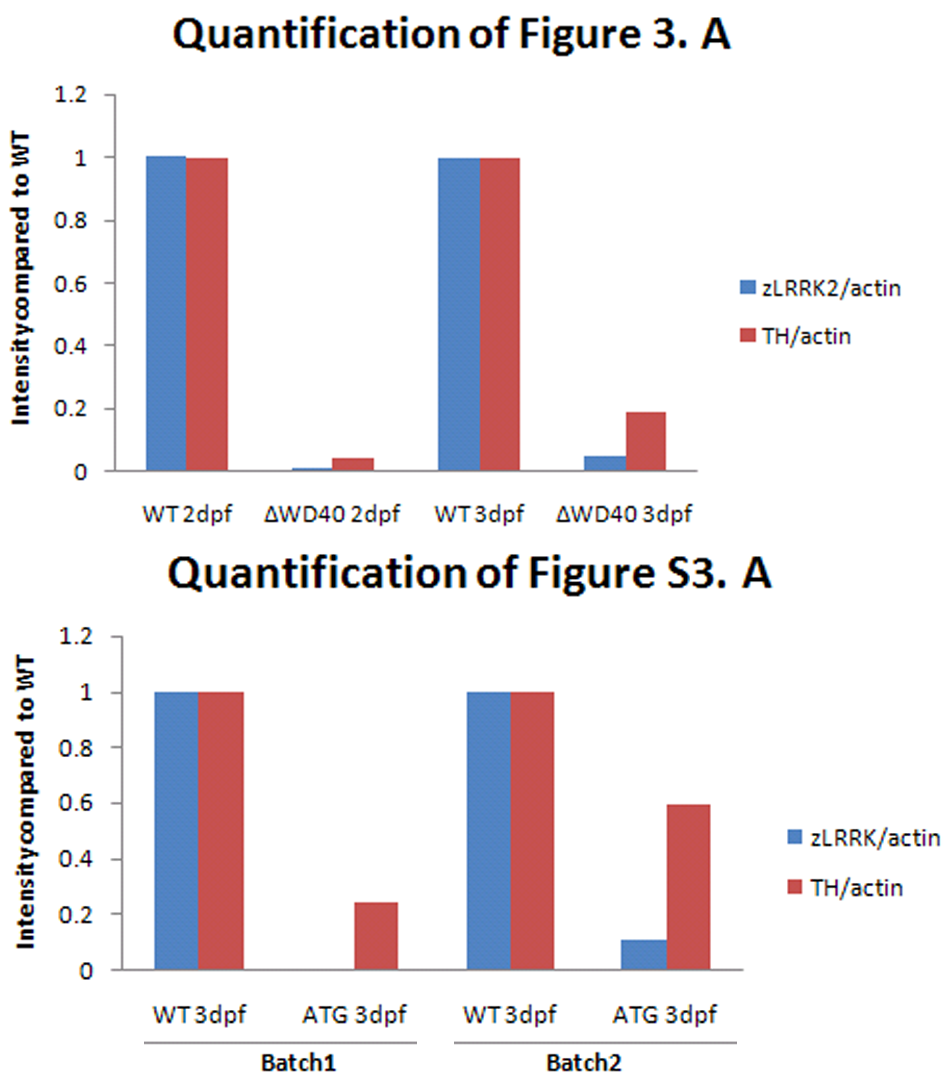

Supplement: Figure S2 — Quantification analysis of the results in Figure 3A and Figure S3A. (0.39 MB TIF) [file pgen.1000914.s002.tif]

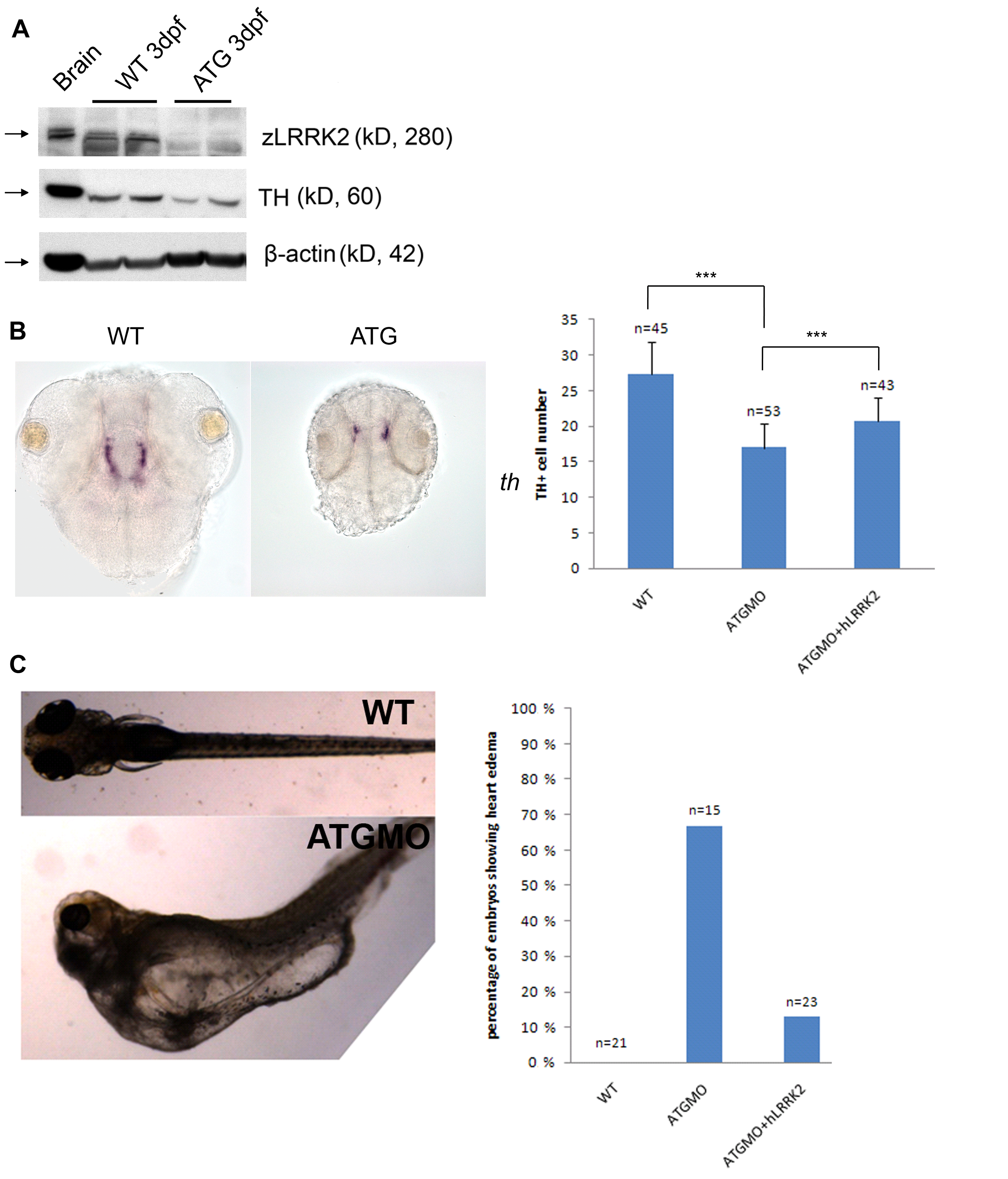

Supplement: Figure S3 — Phenotypes of zLRRK2 ATG morphants. (A) Western blot analysis at 72 hpf showing the strong reduction in expression of zLRRK2 protein as well as the decreased TH protein level in embryos injected with ATG morpholino. The analysis was done in duplicate, and adult brain protein was used as positive control for zLRRK2. (B) WISH analysis at 2 dpf showing retarded brain development and decreased TH expression in an ATG morphant (right). An uninjected sibling is shown for comparison (left). Quantification of the TH+ cell loss in ATG morphant as well as the rescue of ATG morphant with hLRRK2 was shown on the right. Co-injection of hLRRK2 significantly rescue the number of TH+ cells compared to ATG MO alone. ***P<0.001 (unpaired Student's t-test). (C) Heart edema phenotype of ATG morphant and rescue effect of hLRRK2 on ATG morphant. (4.98 MB TIF) [file pgen.1000914.s003.tif]

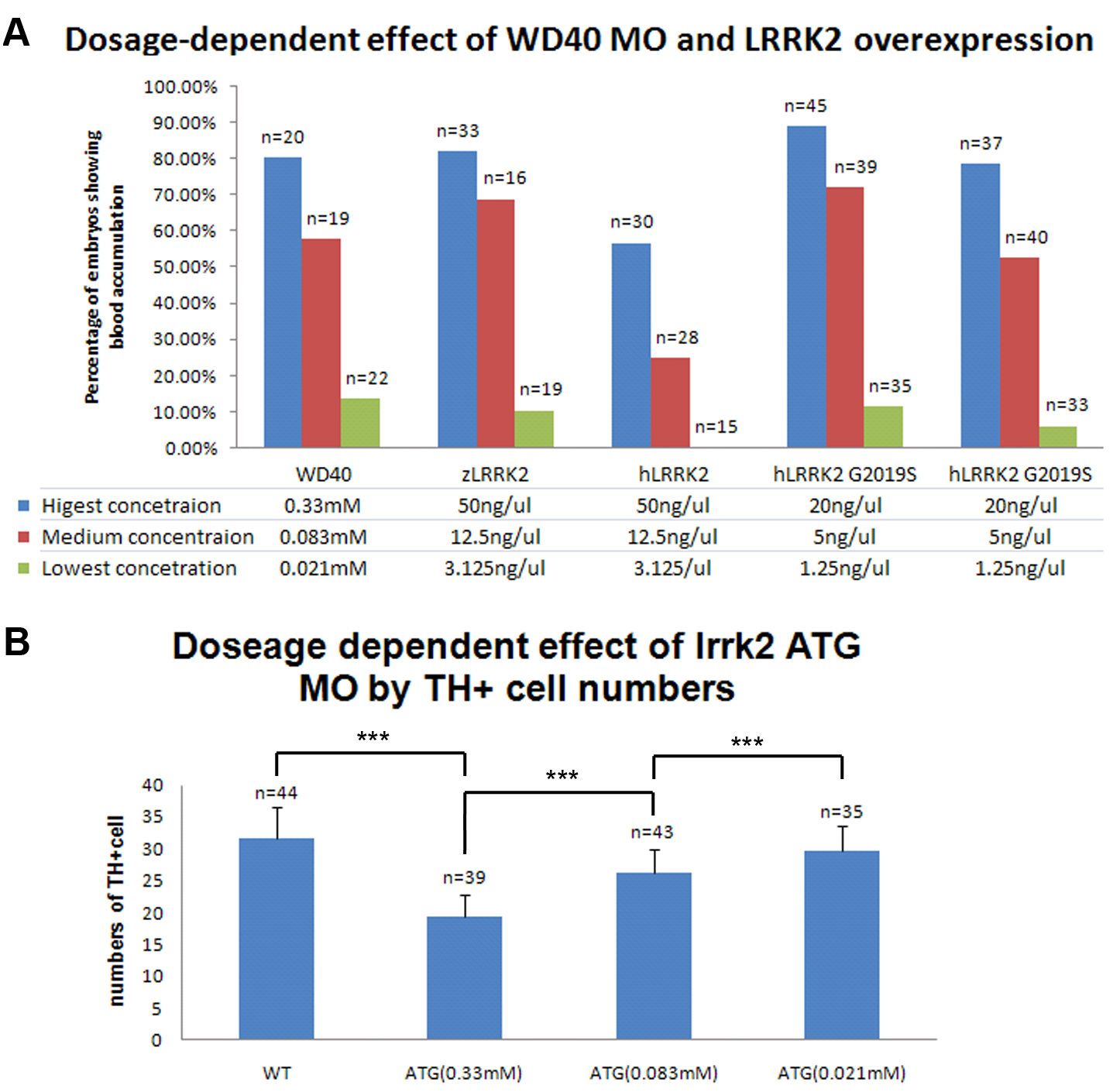

Supplement: Figure S4 — Dosage-dependent effect of morpholinos. (A) Dosage dependent experiment of WD40 morpholino and lrrk2 plasmid (zLRRK2, hLRRK2, hG2019S, and hG2385R) basing on the phenotype of blood accumulation. (B) Dosage dependent experiment of ATG morpholino basing on the quantification of TH+ cell loss. ***P<0.001 (unpaired Student's t-test). (1.01 MB TIF) [file pgen.1000914.s004.tif]

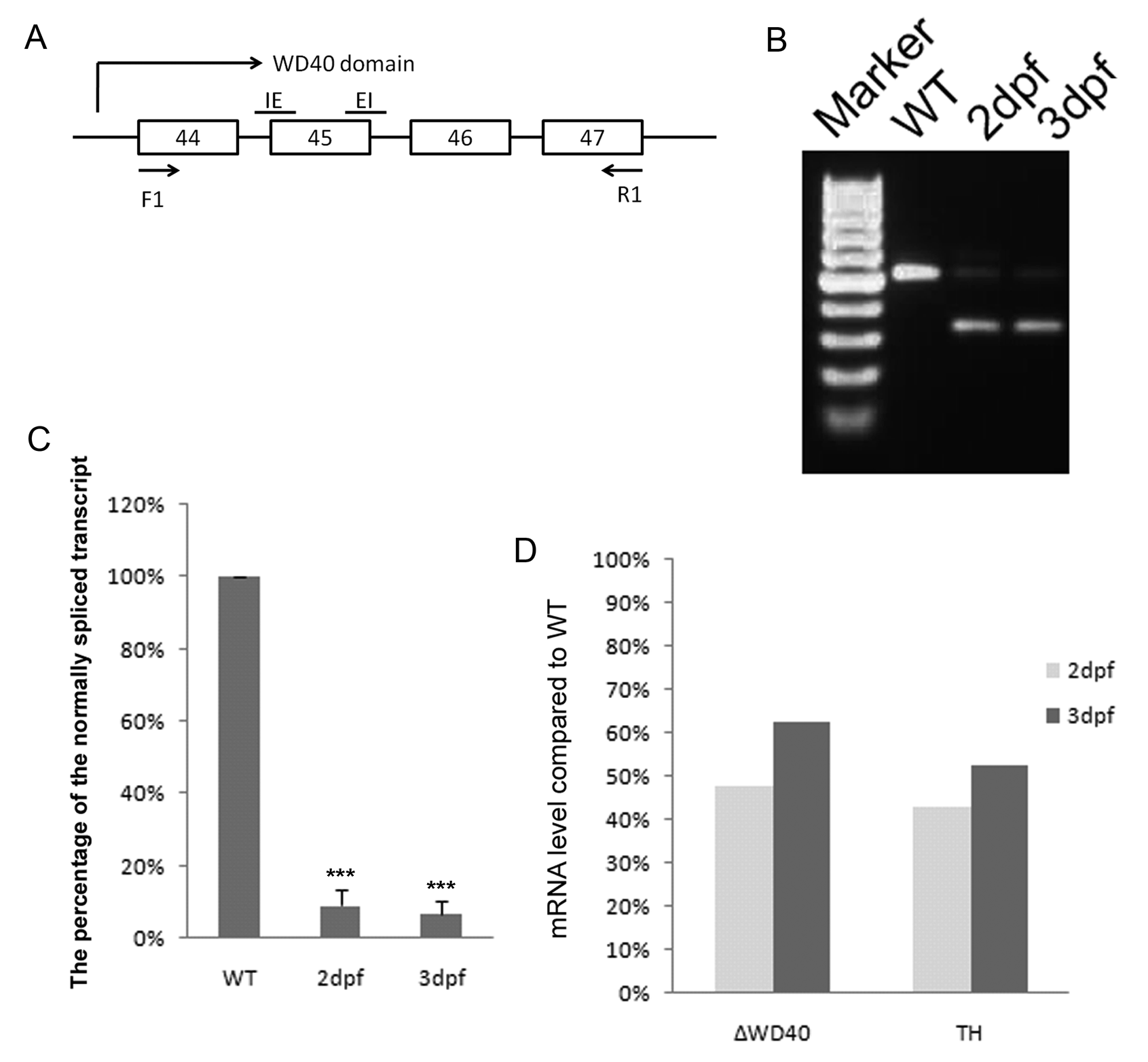

Supplement: Figure S5 — RT-PCR Analysis to confirm the blockage of exon 45 splicing by WD40 morpholino. (A) Schematic representation of the exons 44 to 47 of zLRRK2 to show the morpholinos used for blocking the 45th exon splicing (the track above the exons) as well as the primers (the track below the exons) used in the RT-PCR verification of the splicing blockage effect at 2 dpf and 3 dpf. (B) RT-PCR analysis at 2 and 3 dpf showing that the WD40 morpholino could block the splicing of exon 45, and the major of transcripts were the abnormally spliced one without exon 45 (confirmed by sequence analysis). (C) The percentage of the normally spliced transcript in the total amount of normal and abnormally spliced (without exon 45) transcripts in WT and WD40 morphant at 2 and 3 dpf. The densities of normal and abnormal spliced transcripts (in B) at 2 and 3 dpf were measured, and the percentage of normally spliced transcript was calculated by comparing the density of normal transcript to the total density of both normal and abnormal transcripts (sum of density from normal and abnormal forms). The percentage is presented as mean ± SD from 4 independent experiments. In wild-type embryos, no abnormal splicing forms were detected. ***P<0.001 (unpaired Student's t-test). (D) Quantitative RT-PCR analysis of wild-type controls and WD40 morphants at 2 and 3 dpf, showing that both TH and WT zLRRK2 mRNA levels were reduced to 40%–60% of WT fishes. (0.26 MB TIF) [file pgen.1000914.s005.tif]

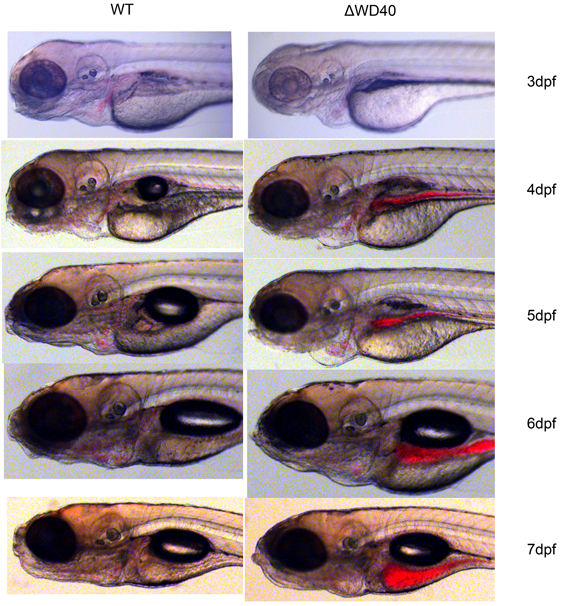

Supplement: Figure S6 — Morphological phenotype of WD40 morphants (From 3 dpf to 7 dpf). Morphants show no significant morphological defects compared to WT fishes except the mild blood accumulation in gut and pronephric duct between the york sac and swimming bladder. (0.91 MB TIF) [file pgen.1000914.s006.tif]

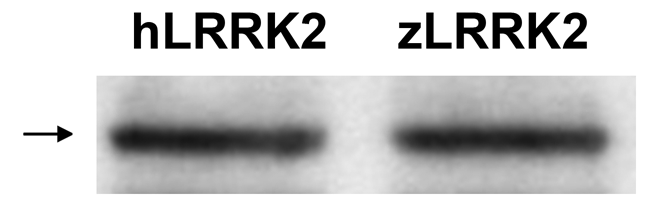

Supplement: Figure S7 — Linearized plasmid harboring either zLRRK2 or hLRRK2 cDNA tagged by Flag can be expression in zebrafish embryos by microinjection. (0.04 MB TIF) [file pgen.1000914.s007.tif]

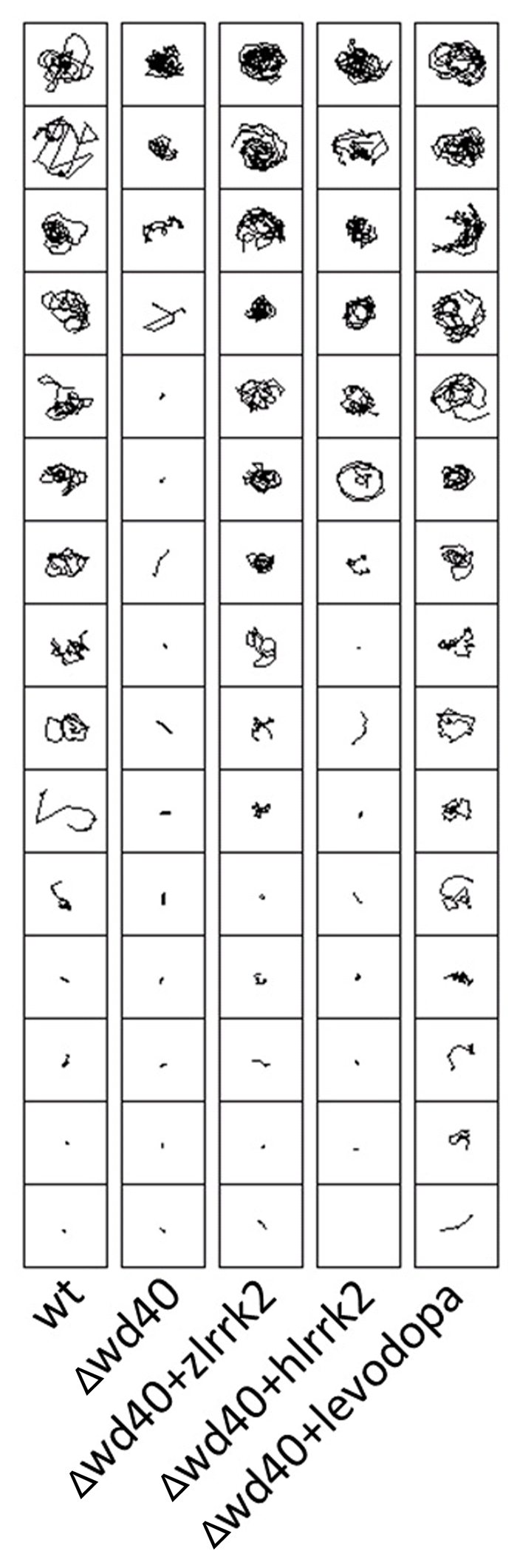

Supplement: Figure S8 — Swimming tracts of 15 fish from each of the five fish pools: wild-type (wt), WD40 morphants (WD40), WD40 morphants co-injected with plasmid harboring either zLRRK2 (WD40+zLRRK2) or hLRRK2 (WD40+hLRRK2) and WD40 morphants treated with levodopa (WD40+levodopa). (0.81 MB TIF) [file pgen.1000914.s008.tif]

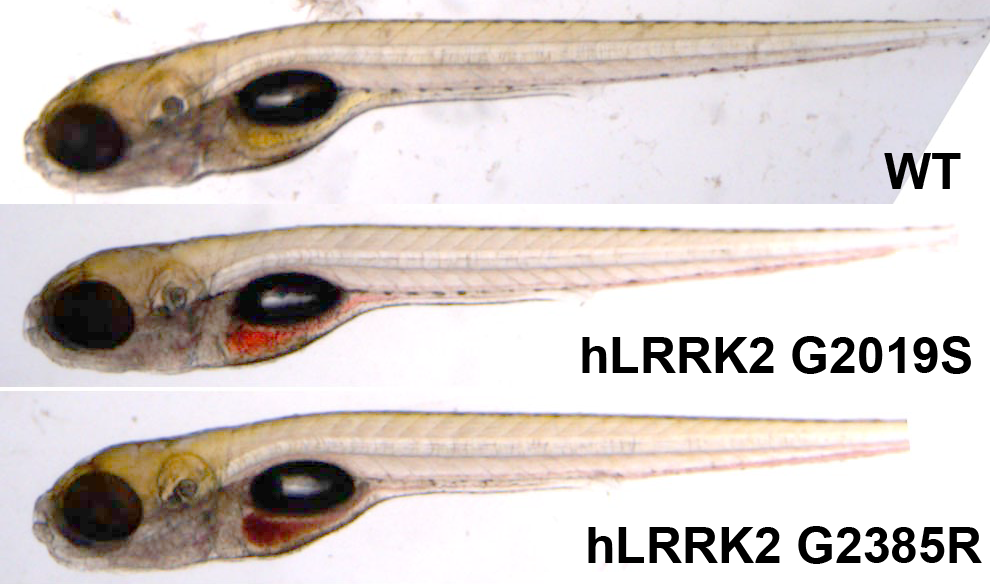

Supplement: Figure S9 — Morphological phenotype of hG2019S and hG2385R overexpression. At 6 dpf, hG2019S and hG2385R overexpression shows no significant morphological defects compared to WT except the blood accumulation in gut and pronephric duct between york sac and swimming bladder. (0.60 MB TIF) [file pgen.1000914.s009.tif]

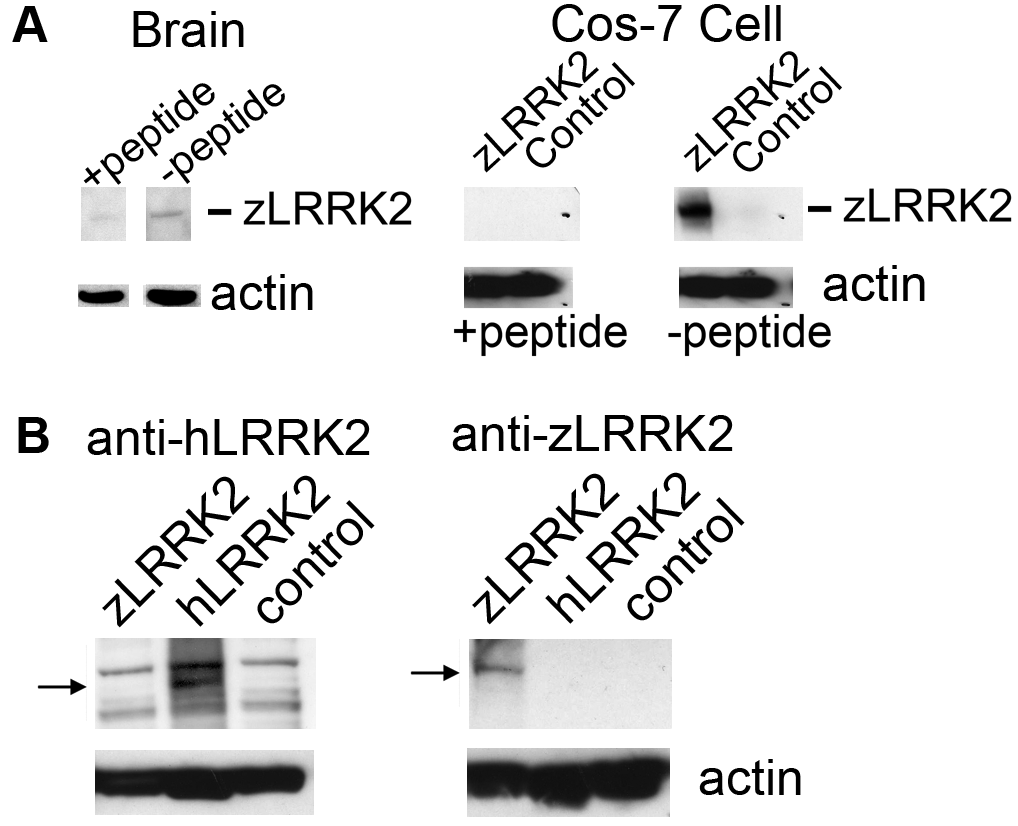

Supplement: Figure S10 — Analysis of zLRRK2 antibody specificity. (A) Specificity of zLRRK2 antibody specificity was verified by western blot analysis. Positive signals could be largely blocked by pre-incubating the anti-zLRRK2 antibody with the neutralizing peptide (+ peptide, left panel). Similarly, the zLRRK2 signal was completely blocked by pre-incubating the anti-zLRRK2 antibody with the neutralizing peptide in the zLRRK2-overexpressing Cos-7 cells (+ peptide, right panel). (B) Western blot analysis showing the specificity of the zLRRK2 antibody against zLRRK2 in Cos-7 cell line. Human LRRK2 and zLRRK2 recombinant proteins were overexpressed in Cos-7 cells separately and detected by anti-hLRRK2 (left panel) and anti-zLRRK2 (right panel), respectively. A positive band (280 kD, arrow) was only detected in hLRRK2-overexpressing cells (hLRRK2, left panel), using anti-hLRRK2 antibody (NOVUS NB 300–268). No obvious band was detected in zLRRK2-overexpressing and untransfected control cells (zLRRK2 and control, left panel); On the other hand, a sharp band (arrow) was detected in zLRRK2-overexpressing cells using the anti-zLRRK2 antibody (zLRRK2, right panel), but not in hLRRK2-overexpressing and untransfected control cells (hLRRK2 and control, right panel). This indicates the anti-zLRRK2 antibody is specifically against zLRRK2 proteins. (0.11 MB TIF) [file pgen.1000914.s010.tif]
