# Supplementary material for: Deletion of the WD40 Domain of LRRK2 in Zebrafish Causes Parkinsonism-Like Loss of Neurons and Locomotive Defect
Source: PLoS Genet. 2010 Apr 22;6(4):e1000914. doi: 10.1371/journal.pgen.1000914 (PMC2858694; doi:10.1371/journal.pgen.1000914)
Supplement: Text S1 — cDNA sequence and exon information of zLRRK2. (0.09 MB DOC) [file pgen.1000914.s011.doc]

aaaatatatgattcataacgcagcctggactacacaggcgccaacatgaccgagatttcct exon1

ccctaaaccgcagagaatcacaccaggaactcaatattagttagtcaggacggcataaat

atggcagagcctctgtgtgaaatctgcggggatgttaatgtgtcaacatggaggatcaga

aagtgcctttaagttcagtttgatgtcagaaactcggagatcatggcagaaatagaggag

M A E I E E

ttgtccatcagactgaagaagctactggtgaggttgaatcttcaggatgggaagcagctg

L S I R L K K L L V R L N L Q D G K Q L

gggactatggtgcagatcatagaggatctcctgttcctgtcccatactgagcactgtgtt exon2

G T M V Q I I E D L L F L S H T E H C V

gagctgtttgcggatcaaaatgtccatgtgcctgtgttgctgaaatgcagcgaaagcgcc

E L F A D Q N V H V P V L L K C S E S A

agcatcaaagttcagcaggttggctggtctctgctgtgtcggctgatggaaatctgtccc exon3

S I K V Q Q V G W S L L C R L M E I C P

aacactctggataatctggccagaccgatggactatgaatttatagatgcacacaagcag exon4

N T L D N L A R P M D Y E F I D A H K Q

attctgaaggtgcttcacgagtaccataataaagatgccaagatgatgatggtggccctg

I L K V L H E Y H N K D A K M M M V A L

agagctctggctctgatgctgaagtcaggtgagattaagatgcaggttctggatgaggag exon5

R A L A L M L K S G E I K M Q V L D E E

gaatgggatgtgttttactcgattctggaggccatgaagtcattcagtgacagagaagaa

E W D V F Y S I L E A M K S F S D R E E

gttcagctacagggatgtacagctttacaaccactgctgcagacagtttctgagtatcac exon6

V Q L Q G C T A L Q P L L Q T V S E Y H

ctggcggagttcatcgagaagaaggaccatgaggtggttttgaacgcgctcggatgcttt

L A E F I E K K D H E V V L N A L G C F

atggacagtgaaaatgtggtacttgaagctctgaaggtcctcataccactggctaatcca

M D S E N V V L E A L K V L I P L A N P

gccagcaatgttgagaccctgatgtccaaaactgtgaaatgccacagtctaacatgtcgg exon7

A S N V E T L M S K T V K C H S L T C R

gctatgaacacgtggctggactccgaggcgatacaggaggctggttgctgtttgctatgg

A M N T W L D S E A I Q E A G C C L L W

aaatttacatcaaagggttactatgatatgctggtgctgaacggtgtccataaagtggcc exon8

K F T S K G Y Y D M L V L N G V H K V A

gtgaaggcatgcgtctcttatcctgacaatgccatattgcagaccgcagctctttcctgt

V K A C V S Y P D N A I L Q T A A L S C

ctgagtgcccttgcggagtgtattgtacaaaatggaggactggatgaagagtggaatgag exon9

L S A L A E C I V Q N G G L D E E W N E

gaggacgaggaggaacagaaagttctggtaaagaaagaagccgctcaggcggaggagatg

E D E E E Q K V L V K K E A A Q A E E M

ctaatctggagagaagcctgttatacggcttttgaaagacatgctgaagacgtcaaagta

L I W R E A C Y T A F E R H A E D V K V

caggaagctgcatgctggactttaaatagtttgcttctccattgcaacacatccaaccat exon10

Q E A A C W T L N S L L L H C N T S N H

gtggaactggaaggaaggcctcctcttcatacgctcatcatggctgcgatgcttttgcac exon11

V E L E G R P P L H T L I M A A M L L H

tcatccagcgtgaaggttttccaagctgcttcctctacactgcgcacgctaatacagcgc

S S S V K V F Q A A S S T L R T L I Q R

cactgcaggataagatctccactactctcaaatggcattcatttcaacatcgtggagctg exon12

H C R I R S P L L S N G I H F N I V E L

atgcggaaacaccctaactccagcgctgtgtgtgaaagtgcctgcaaactaattcacaca

M R K H P N S S A V C E S A C K L I H T

ctcttccagggagcgagggccagtctggacgacgggttcctgattctcagtcagatcctc exon13

L F Q G A R A S L D D G F L I L S Q I L

atcgctctgaagactcacaccttccttcctgaagttcagctggagggtctgcgagcaagt

I A L K T H T F L P E V Q L E G L R A S

ttagttcttcttaatccagacaggagtttgagagagcatggcgtctcggtggcagatcct exon14

L V L L N P D R S L R E H G V S V A D P

gatatggtggacgtttctctacaggtgttgaagaaccagtgtgtcctagagggagctcac

D M V D V S L Q V L K N Q C V L E G A H

actgtctatctgcaagcacttaataggtttatcagcagcgagtcgattcaggagtgtggt exon15

T V Y L Q A L N R F I S S E S I Q E C G

ctcggcgtcctggcagcgctagctgacagttcgggagctgtagatctgatgtgccagcag

L G V L A A L A D S S G A V D L M C Q Q

ggggcgatagacaccgttctgcacacgctacagatgtttccacaggagcgcgatattcac exon16

G A I D T V L H T L Q M F P Q E R D I H

tactggggtctgagtctcctcttccacctgatctctaagaagaaactgtctcgtatgatg

Y W G L S L L F H L I S K K K L S R M M

gttcctgtgctggcgtctgtgttggtcagctctgtgcggaaacacaaagaggactcagtc

V P V L A S V L V S S V R K H K E D S V

atgctgctaaagggtctacaggtggtgtggaagttgttggacacctgctcgagtgccgct exon17

M L L K G L Q V V W K L L D T C S S A A

gtgtggctgcagaaagaagcgttcgagaaggagatcttccagatcctgcgggagaacacg

V W L Q K E A F E K E I F Q I L R E N T

gccgatcagcgcagagatccgctgcagggaatgagctgcctgtgcttgtctaagatggtg exon18

A D Q R R D P L Q G M S C L C L S K M V

atggatggagagatcctgtacgctctgctggagagagcgtgtgaggatggagacgtggac

M D G E I L Y A L L E R A C E D G D V D

atggcagagtgtctgatccagctgggcgccgacgtcaacaagaaaaccaagagcgactca

M A E C L I Q L G A D V N K K T K S D S

ctcctatatcaggcgtgtgatcgcggggctcccctgtctctgctggagcttctggtttct exon19

L L Y Q A C D R G A P L S L L E L L V S

tcaggcgttcatgagcagcatctgcgtggagctctgagtgtgtgcgtgcggaggtcggat

S G V H E Q H L R G A L S V C V R R S D

gatcctgctgtcactctgatcctccgccgtctgggacttgaccacaccaacagcgccctg

D P A V T L I L R R L G L D H T N S A L

tgtctcggcagcgtccgcatcggacacatgaaggcctcgtggatcagcgctcttctgtct

C L G S V R I G H M K A S W I S A L L S

gagcgcaggagtcaatctacaaacacgcactacagcagtaaaggtcagcgtttggccaga exon20

E R R S Q S T N T H Y S S K G Q R L A R

cagatctcgcagctccagaggaagaaggggattgtgggagttagcagattatcgagtgat

Q I S Q L Q R K K G I V G V S R L S S D

gtcagcacatcaggatatttcaccgatgaggaaagtgacgactcgcacatttccttagag

V S T S G Y F T D E E S D D S H I S L E

gacagcttggtctttatgttcgatgagttggagagcgacggcagtgacggccctccacat exon21

D S L V F M F D E L E S D G S D G P P H

ggattcatgctaatcagtgattctccggaagttagcaggagacccgtctggaggcgtcgc

G F M L I S D S P E V S R R P V W R R R

agccacagccgacgtaccagttcagagggctaccatggagaggcggatccaagtgtccct exon22

S H S R R T S S E G Y H G E A D P S V P

gtgcagaagcacgccaactcccacaacagcagaggtcagggtttctctgagagtttctcc exon23

V Q K H A N S H N S R G Q G F S E S F S

agtcctgtggtcctggataaagacccggtgcgtctgctggatctgtctgggaatgagctg

S P V V L D K D P V R L L D L S G N E L

aacgatctctcctgtctgacggatctgaactctctcaagaaaccgattgaaaaccttcat

N D L S C L T D L N S L K K P I E N L H

Cggctcgatttgagcggcaacaatctgtcacagtttcccagcattctctgtcagagtttg exon24

R L D L S G N N L S Q F P S I L C Q S L

cgcagtctgactcgtctggatctgcagggaaaccatctgcagtgtttgccatctgaactg

R S L T R L D L Q G N H L Q C L P S E L

ctcagtttaccagcgctccacacactgaacgtgtcccgcaactgcatcggtccgctcctg

L S L P A L H T L N V S R N C I G P L L

cagctggagcccggcgtctgcagtccagccctgcggcgcctcaacctgtccttcaaccag

Q L E P G V C S P A L R R L N L S F N Q

atcactgtctgcccattccagctgcgcagcgccacccagaggctggaggagctctcactg

I T V C P F Q L R S A T Q R L E E L S L

gagggaaatcagatctccgaactctccctcccgctctgtctggctgagctgaaggtttta exon25

E G N Q I S E L S L P L C L A E L K V L

gacgtcagtaagaatcaggttaaaatagtttcagacaacttccttgctgaatgcctcaag

D V S K N Q V K I V S D N F L A E C L K

atggaaactttcatcgcttctgttaaccagatcagttcactgccacaccttccctccaaa exon26

M E T F I A S V N Q I S S L P H L P S K

ataaccaccgtgaaattatcacacaacactttcaccagcgttcctgaaatagtcatcaac

I T T V K L S H N T F T S V P E I V I N

ttgccatgcttgcgttcggtggacatgaggaataacagtgttggcgttctgccgggtccg exon27

L P C L R S V D M R N N S V G V L P G P

tctgtctggctgagtgtgaacctcagagagctgatgttcagccataatctcatctccgca

S V W L S V N L R E L M F S H N L I S A

ctcgacctcagcggccccgtgtacaagtgggcccgactggagaaactgcacttgagcttc

L D L S G P V Y K W A R L E K L H L S F

aatagactcactgagattcctcctcagatcgggatgctggaggacctgacgtctctggat exon28

N R L T E I P P Q I G M L E D L T S L D

gtgagccataatgagggtctgcgctcgttcccggatgagatggggaagctggtgcatctg

V S H N E G L R S F P D E M G K L V H L

tgggatctgcctctggatggcctgcagctccagctggacctcaaacacatcgggagcaaa

W D L P L D G L Q L Q L D L K H I G S K

acaaaagacatcatcaggttcctgcagcagcgtctgaagaaggccgtcccgtatcaccgc exon29

T K D I I R F L Q Q R L K K A V P Y H R

atgaagctgatggtgttggggggcaccggcagcggaaagagctctttaattcagcagctg

M K L M V L G G T G S G K S S L I Q Q L

atgagactcaggcgctcacaatggagatcagatccaggcgtcagcattcgggactggccc

M R L R R S Q W R S D P G V S I R D W P

gtcaggagcaaagacaagaggaacatgatgctgaacgtctgggagttctcaggtggtgaa exon30

V R S K D K R N M M L N V W E F S G G E

gagtgcagtgggattcaccctcacttcatgagctctagagctgtctatctagtgctctac

E C S G I H P H F M S S R A V Y L V L Y

gacctcagtaaaggagccagcgagatccactccatcaaaccctggctctttaacattaag

D L S K G A S E I H S I K P W L F N I K

gctgtagcgggtcaatgtccagtgattgtggtgggaactcatgcagatttgtgtgaagag exon31

A V A G Q C P V I V V G T H A D L C E E

cgccacctacaggagtgtttgctgaagctgcagaaagagcttcagtctcagcccgggttt

R H L Q E C L L K L Q K E L Q S Q P G F

ccggcgatcagagaaaaccacgtgttgagtgcctgcgaggagtccgagtcactcggccgg

P A I R E N H V L S A C E E S E S L G R

ctacgcaaagccatctacagagaactcatcgggttcaagatccagggccagccggtgatg exon32

L R K A I Y R E L I G F K I Q G Q P V M

ggtcagctagttccagactgttatgtggagctggagaagagactcctgcaggagagatca

G Q L V P D C Y V E L E K R L L Q E R S

tgcgctccggctgatttcccagtgctcagacacagcagactgatggagatcctgcaggag

C A P A D F P V L R H S R L M E I L Q E

acgcagttacagctggaggagggggagttacctcacgctatacacttcctcagcgaagcc

T Q L Q L E E G E L P H A I H F L S E A

ggtgtcttgctgcactttgatgatccagttcttcagctgaaggatctgtacttcattgac exon33

G V L L H F D D P V L Q L K D L Y F I D

Ccacagtggttttgtaggatcatctcacagacgctgtctctgaaaagcagcggtccatgg exon34

P Q W F C R I I S Q T L S L K S S G P W

gacagcacgaaaggagtggtgcagcgctccaccgtggagaaatttgtggagaaaagccgc

D S T K G V V Q R S T V E K F V E K S R

tgtttcccaaaggatcacatgattcaatacttcaaactgttggagaagtttcagatagcc

C F P K D H M I Q Y F K L L E K F Q I A

ctgccatttgaccatgaccagctactcattcctagcagtctgtcagaccacaggccagtg exon35

L P F D H D Q L L I P S S L S D H R P V

attgagcttcctcactgtgagaattcagaagtgatcattcgactatatgagatgccatat

I E L P H C E N S E V I I R L Y E M P Y

tttccgatgggctactggcccagacagatcagccgtctgctggaagtgtctgcattcctg

F P M G Y W P R Q I S R L L E V S A F L

ctctatggcagagaaaaagcattaaaaccaaaccggatctactggaggaagggcatctat exon36

L Y G R E K A L K P N R I Y W R K G I Y

ttgagctggtctgctgaagcttactgtctggttgaggctttgactttagaagaaaaccca

L S W S A E A Y C L V E A L T L E E N P

gctagtttcatcaagatcaccgttccatgctctcgcaaaggccgtgtgttgtttgggcag exon37

A S F I K I T V P C S R K G R V L F G Q

gtggtggaccacattgactctctgctggaggaatggtttccaggccttctcaccactgac

V V D H I D S L L E E W F P G L L T T D

attcatggcactggagagacgctgctgaagaaatgggctctgtacagcttcagcgatgga

I H G T G E T L L K K W A L Y S F S D G

caaaactgccagaagatgctactggaagacctgctctccaacaccaacgcagatggtcta exon38

Q N C Q K M L L E D L L S N T N A D G L

ctggtgaaccccgaggaccccagctgtactttacccatctctcagatttctccagacctg

L V N P E D P S C T L P I S Q I S P D L

gtactgagtgaccagccatccagcaccatactggacccagagcagctggagatggagctc

V L S D Q P S S T I L D P E Q L E M E L

actgcagagtatatgttgggggatggaggttttggctcggtttataaagctgtgtacaag exon39

T A E Y M L G D G G F G S V Y K A V Y K

aatgaagaagttgcggtcaagatttttaataaacatgcgtctgcgctgtacgttcaccgg

N E E V A V K I F N K H A S A L Y V H R

ctggtacgacaggaactggcagtactcggcagactgtgtcatcctagtttggtgggtctt exon40

L V R Q E L A V L G R L C H P S L V G L

ttggctgctggttgtaacccacatatacttgtgatggagttggctccatatggctctctg

L A A G C N P H I L V M E L A P Y G S L

gactcgctgtttgagcgtgaaaatggcagcttaagtcgcaaattacagcacaggatcgca

D S L F E R E N G S L S R K L Q H R I A

ctacatgtggctgatggtctcaaatatttgcactcatctatgatcatctaccgagacctg exon41

L H V A D G L K Y L H S S M I I Y R D L

aagccgcacaatgttctgctgttcaacctgaagacggacgctgagatcgtcgctaaaatc

K P H N V L L F N L K T D A E I V A K I

acagactatggcatcgcgcagtactgctgcagcatgggcgtccgcagctctgaaggaaca

T D Y G I A Q Y C C S M G V R S S E G T

ccaggttttcgtgccccggaggtggctcgaggaaacgtcatctacaacgtccaggcggat exon42

P G F R A P E V A R G N V I Y N V Q A D

gtttattcgttcggtctgctactgtatgatctgttaacatacggcgagcgcatctcagat

V Y S F G L L L Y D L L T Y G E R I S D

ggcatgaagtttcccagtgagtttgatgaagtggccgtgcagggcaaactaccagatcca exon43

G M K F P S E F D E V A V Q G K L P D P

gtgaaggattacggctgttctccatggcctgagattgagtctcttatgagggaatgcatg

V K D Y G C S P W P E I E S L M R E C M

agagaaaacccccaggacaggccgacgtctgctcaggtgtttgatcgtctgaactctgca exon44

R E N P Q D R P T S A Q V F D R L N S A

gagatgttgtgtctgacgcgagagctgaatgttgtgggttttccaggcgagtgttttgtc

E M L C L T R E L N V V G F P G E C F V

gtgtccaattcaggcggagctgcaaacggaggtaaaaaccctcatgtgtggatcggtggt

V S N S G G A A N G G K N P H V W I G G

ggcagcagcagccagaaactgggctgtgtgactgcggtggatctggagaccggcgggagt

G S S S Q K L G C V T A V D L E T G G S

ttgaaccaggagctggaccgcagccctatcctctgcatggtgatcatcagagctgcagac exon45

L N Q E L D R S P I L C M V I I R A A D

tcgtgcagtgactggttagtggctggttcagagtccggttcgctctctatcatggacacc

S C S D W L V A G S E S G S L S I M D T

attaatgcgaaagtcctgcaccgtctgaagagtgtgaaggactctgtgacgtctctgtac

I N A K V L H R L K S V K D S V T S L Y

tttcacaccgagctccagcacagatgtctgaagagttatcttttggtgggcacagcagac exon46

F H T E L Q H R C L K S Y L L V G T A D

gggacactggtcatttatgaagattcagccctgaagctggagaacggtggtccggtgaag exon47

G T L V I Y E D S A L K L E N G G P V K

acgctggaggttggagatgtgaacactccgctgatgtgtctcggcccatccagtcatcct

T L E V G D V N T P L M C L G P S S H P

caggagcgcagatcgctgtgggccgcctgtgggaccaggatcatcttattcactgttgaa

Q E R R S L W A A C G T R I I L F T V E

Ttcgacgtctgtagaagcattgacaccaaacccaaaccgctcttcccactgcaggctcgg exon48

F D V C R S I D T K P K P L F P L Q A R

gtcagcggggaggcttgtatatcacggttagcggtggacaaacacgtgtatgtgagtaaa

V S G E A C I S R L A V D K H V Y V S K

accggaggccacactgttgaggtctgggacaagaagactgagaggatggtgaacctcatc

T G G H T V E V W D K K T E R M V N L I

gactgcatgcagttactcggattaagttccaccagaaagcccaaagttcattcagaggac exon49

D C M Q L L G L S S T R K P K V H S E D

cagtctagaccgatggtgccgtcgttggttgtgaaagccttattggtccagcacagtggg

Q S R P M V P S L V V K A L L V Q H S G

actctgtggatcgggacgagagcgggacacattttgttggtggaggtttccagttgtcat

T L W I G T R A G H I L L V E V S S C H

cttttacagaccatcaaccctcactgccactccatacgctgcatgagctccatacttcta

L L Q T I N P H C H S I R C M S S I L L

gacacactaaaccgaaagaatgtcattctagtattgggtagacggcagcgcatacatcta exon50

D T L N R K N V I L V L G R R Q R I H L

gagcagctcaaaacacaatcaggcgaggactccgtgctgacgctctggagcagttctctt exon51

E Q L K T Q S G E D S V L T L W S S S L

cctctggaggctcgagatctgatgcgacactgtgagctccgagacaaaaccacccgcaga

P L E A R D L M R H C E L R D K T T R R

atgagagaaacgctgcttaattaaacaccctctccatacaaaaacatcagaataaatccg

M R E T L L N -

ccatcgacaactccgtcaaactaaacagcatcaggtgaatttgtgcacaaaaaaaaacaa

gtccagcgtgtagctgagcgcggaaatgctttaaaaaggggatggttcactcaaaaatga

aaactacggcattaattattagtcacaaacctgttaaactgactttcttcagttgaataa

gagaagatatttggaagaatgctgaaaacctgtaaccattgacttccatgttgtttttca

tactatggaagtcaatagttacagcatttttcaaaatctcctttcgggttcaacagaaaa

cagaaactcttaacaggtttggaagcacttgagggagactaactagactaatacaattac

tagctcataaaaaatgtgctgaaacaactgaaattctcgggaaaacttgcttttttactt

gcgttttatgttcaacacactaagttaatcgatttgtgtctggacaacttgaacgtatgg

tagttttgtttacagtgtatcgagtacatttcattttcgggtgaactgtccctttaagag

tgtgtttgaaatgtctccgagcagcttttaatgcgaccacagctgtactagtcacgaaaa

gggacccgaaatccatttcagcttctttaaatagccaagggaaactttcattgtggtgaa

tatatcataaatgtagctcgtatatctcagatgtagctgtgattcggattgtatggaagc

tcatatgaggacggaaccaaaacagcacttgattatgttagccttattattttgtagtat

ttatttattattggtttaaagccatgtgaagaggaggcggccatatacaatgcatctgtg

atgatgcattacagcagtcatcattctttcccccattaatggataatacattaataatga

aagcattaatgctggttgtgagattgcgaatgaaggcaatgtaatacatcacatgatgat

atcatatggtttgtggagacaatctgttgctgagacttgagcgatttacgttaagctaat

aaaccacactacacaatttatgatttattataatcatgttatttatgatgtccgcagcga

gccagtgatttattttttttattattaattaacttcattaagttgagcacaaacattata

actggagcataagccatagattaaaagctatgattatgattattttggttactgaaatga

cccttcactgttacatttttgtgatttgtgggaatgtaattattattatgatttataatc

gaatgctgcatgccacgtatgtttttaataaagctgccattttgtatgat

Blasting the above cDNA sequence against the genome sequence at the UCSC Genome Browser (version Dec 2008) revealed two mismatches of 36 bp and 17 bp (below). To further investigate the mismatches, we sequenced the genomic sequence fragment of 1391 bp, covering the two mismatches, of the fish strain used in this study. The analysis revealed the same mismatches between the genome sequence of the fish strain used in this study and the one at the UCSC Genome Browser (version Dec 2008). Therefore, cDNA sequence can fully match the genome sequence of the fish strain used in this study.

CDNA CGAGACAAAACCACCCGCAGAATGAGAGAAACGCTGCTTAATTAAACACCCTCTCCATAC 60

UCSC_Genome CGAGACAAAACCACCCGCAGAATGAGAGAAACGCTGCTTAATTAAACACCATCTCCACAC 60

************************************************** ****** **

CDNA AAAAACATCAGAATAAATCCGCCATCGACAACTCC--GTCAAACTAAACAGCATCAGGTG 118

UCSC_Genome AAAAACATCAGAATAAATCCGCCATCGACAACTCCCTGTCAAACTAAACAGCATCAGGTG 120

*********************************** ***********************

CDNA AATTTGTGCACAAAAAAAAACAAGTCCAGCGTGTAGCTGAGCGCGGAAATGCTTTAAAAA 178

UCSC_Genome AATTTGTGCACAAAAAACCCCAAGTCCAGCGTGTAGCTGAGCGTGGAAATGCTTTAAAAA 180

***************** *********************** ****************

CDNA GGGGATGGTTCACTCAAAAATGAAAACTACGGCATTAATTATTAGTCACAAACCTGTTAA 238

UCSC_Genome GGGGATGGTTCACTCAAAAATGAAAACTACGGCATTAATTATTAGTCACAAACCTGTTAA 240

************************************************************

CDNA ACTGACTTTCTTCAGTTGAATAAGAGAAGATATTTGGAAGAATGCTGAAAACCTGTAACC 298

UCSC_Genome ACTGACTTTCTTTAGTTGAATAAGAGAAGTCAATGGTTACAGGTTTTCAGCATTCTTCCA 300

************ **************** * * * * * * * * * *

CDNA ATTGACTTCCATGTTGTTTTTCATACTATGGAAGTCAATAGTTACAGCATTTTTCAAAAT 358

UCSC_Genome AATATCTTCTATGTTGTTTTTCATACTATGGAAGTCAATAGTTACAGCATTTTTCAAAAT 360

* * **** **************************************************

CDNA CTCCTTTCGGGTTCAACAGAAAACAGAAACTCTTAACAGGTTTGGAAGCACTTGAGGGAG 418

UCSC_Genome CTCCTTTCGGGTTCAACAGAAAACAGAAACTCTTAACAGGTTTGGAACCACTTGAGGGAG 420

*********************************************** ************

CDNA ACTAACTAGACTAATACAATTACTAGCTCATAAAAAATGTGCTGAAACAACTGAAATTCT 478

UCSC_Genome AGTAACTAGACTAATACAATTACTAACTCATAAAAAATGTGCTGAAACAACTGAAATTCT 480

* *********************** **********************************

CDNA CGGGAAAACTTGCTTTTTTACTTGCGTTTTATGTTCAACACACTAAGTTAATCGATTTGT 538

UCSC_Genome CGGGAAAACTTGCTTTTTTACTTGCGTTTTATGTTCAACACACTAAGTTAATCGATTTGT 540

************************************************************

CDNA GTCTGGACAACTTGAACGTATGGTAGTTTTGTTTACAGTGTATCGAGTACATTTCATTTT 598

UCSC_Genome GTCTGAACAACTTGAATGTATGGTAGTTTTGTTTACAGAGTAGCGAGTACATTTCATTTT 600

***** ********** ********************* *** *****************

CDNA CGGGTGAACTGTCCCTTTAAGAGTGTGTTTGAAATGTCTCCGAGCAGCTTTTAATGCGAC 658

UCSC_Genome CGGGTGAACTGTCCCTTTAAGAGTGTGTTTAAAATGTCTCCGAGCAGCTTTTAATGCGAC 660

****************************** *****************************

CDNA CACAGCTGTACTAGTCACGAAAAGGGACCCGAAATCCATTTCAGCTTCTTTAAATAGCCA 718

UCSC_Genome CACAGCTTTACTAGTCACGAAAAGGGACCCGAAATCCATTTCAGCTTCTTTAAATAGCCA 720

******* ****************************************************

CDNA AGGGAAACTTTCATTGTGGTGAATATATCATAAATGTAGCTCGTATATCTCAGATGTAGC 778

UCSC_Genome AGGGAAATTTTCATTGTGGTGAATATATCATAAATGTAGCTCGTA--TCTCAGATGTAGC 778

******* ************************************* *************

CDNA TGTGATTCGGATTGTATGGAAGCTCATATGAGGACGGAACCAAAACAGCACTTGATTATG 838

UCSC_Genome TGTGATTCGGATTGTATGGAAGCTCATATGAGGACGGAACCAAAACAGCACTTGATTATG 838

************************************************************

CDNA TTAGCCTTATTATTTTGTAGTATTTATTTATTATTGGTTTAAAGCCATGTGAAGAGGAGG 898

UCSC_Genome TTAGCCTTATTATTTTGTAGTATTTATTTATTATTGGTTTAAAGCCATGTGAAGAGGAGG 898

************************************************************

CDNA CGGCCATATACAATGCATCTGTGATGATGCATTACAGCAGTCATCATTCTTTCCCCCATT 958

UCSC_Genome CGGCCATATACAATGCATCTGTGATAATGCATTACAGCAGTCATCATTCTTTCCCCCATT 958

************************* **********************************

CDNA AATGGATAATACATTAATAATGAAAGCATTAATGCTGGTTGTGAGATTGCGAATGAAGGC 1018

UCSC_Genome AATGGATGATACATTAATAATGAAAGCATTAATGCTGGTTGTGAGATTGTGAATGAAGGC 1018

******* ***************************************** **********

CDNA AATGTAATACATCACATGATGATATCATATGGTTTGTGGAGACAATCTGTTGCTGAGACT 1078

UCSC_Genome AATGTAATATATCACATGATGACATTATATGGTTTGTGGAGACAATCTATTGCTGAGACT 1078

********* ************ ** ********************** ***********

CDNA TGAGCGATTTACGTTAAGCTAATAAACCACACTACACAATTTATGATTTATTATAATCAT 1138

UCSC_Genome GGAGCGATTTACGTTAAGCTAATAAACCACACTACACAATTTATGATTTATTATAATCAT 1138

***********************************************************

CDNA GTTATTTATGATGTCCGCAGCGAGCCAGTGATTTATT------TTTTTTATTATTAATTA 1192

UCSC_Genome GTTATTTATGATGTCCGCAGCGAGCCTTTTTTTTATTATTATTTTTTTTATTATTAATTA 1198

************************** * ****** *****************

CDNA ACTTCATTAAGTTGAGCACAAACATTATAACTGGAGCATAAGCCATAGATTAAAAGCTAT 1252

UCSC_Genome ACTTCATGAAGTTGAGCACAAACATTATAACTGGAGCATAAGCCATAGATTAAAAGCTAT 1258

******* ****************************************************

CDNA GATTATGATTATTTTGGTTACTGAAATGACCCTTCACTGTTACATTTTTGTGATTTGTGG 1312

UCSC_Genome GATTATGATTATTTTGGTTACTGAAATGACCCTTCACTGTTACATTTTTGTGATTTGTGG 1318

************************************************************

CDNA GAATGTAATTATTATTATGATTTATAATCGAATGCTGCATGCCACGTATGTTTTTAATAA 1372

UCSC_Genome GAATGTAATTATTATTATGATTTATAATCGAATGCTGCATGCCACGTATGTTTTTAATAA 1378

************************************************************

CDNA AGCTGCCATTTTGTATGAT 1391

UCSC_Genome AGCTGCCATTTTGTATGAT 1397

*******************

Our_Genome CGAGACAAAACCACCCGCAGAATGAGAGAAACGCTGCTTAATTAAACACCCTCTCCATAC 60

UCSC_Genome CGAGACAAAACCACCCGCAGAATGAGAGAAACGCTGCTTAATTAAACACCATCTCCACAC 60

************************************************** ****** **

Our_Genome AAAAACATCAGAATAAATCCGCCATCGACAACTCC--GTCAAACTAAACAGCATCAGGTG 118

UCSC_Genome AAAAACATCAGAATAAATCCGCCATCGACAACTCCCTGTCAAACTAAACAGCATCAGGTG 120

*********************************** ***********************

Our_Genome AATTTGTGCACAAAAAAAAACAAGTCCAGCGTGTAGCTGAGCGCGGAAATGCTTTAAAAA 178

UCSC_Genome AATTTGTGCACAAAAAACCCCAAGTCCAGCGTGTAGCTGAGCGTGGAAATGCTTTAAAAA 180

***************** *********************** ****************

Our_Genome GGGGATGGTTCACTCAAAAATGAAAACTACGGCATTAATTATTAGTCACAAACCTGTTAA 238

UCSC_Genome GGGGATGGTTCACTCAAAAATGAAAACTACGGCATTAATTATTAGTCACAAACCTGTTAA 240

************************************************************

Our_Genome ACTGACTTTCTTCAGTTGAATAAGAGAAGATATTTGGAAGAATGCTGAAAACCTGTAACC 298

UCSC_Genome ACTGACTTTCTTTAGTTGAATAAGAGAAGTCAATGGTTACAGGTTTTCAGCATTCTTCCA 300

************ **************** * * * * * * * * * *

Our_Genome ATTGACTTCCATGTTGTTTTTCATACTATGGAAGTCAATAGTTACAGCATTTTTCAAAAT 358

UCSC_Genome AATATCTTCTATGTTGTTTTTCATACTATGGAAGTCAATAGTTACAGCATTTTTCAAAAT 360

* * **** **************************************************

Our_Genome CTCCTTTCGGGTTCAACAGAAAACAGAAACTCTTAACAGGTTTGGAAGCACTTGAGGGAG 418

UCSC_Genome CTCCTTTCGGGTTCAACAGAAAACAGAAACTCTTAACAGGTTTGGAACCACTTGAGGGAG 420

*********************************************** ************

Our_Genome ACTAACTAGACTAATACAATTACTAGCTCATAAAAAATGTGCTGAAACAACTGAAATTCT 478

UCSC_Genome AGTAACTAGACTAATACAATTACTAACTCATAAAAAATGTGCTGAAACAACTGAAATTCT 480

* *********************** **********************************

Our_Genome CGGGAAAACTTGCTTTTTTACTTGCGTTTTATGTTCAACACACTAAGTTAATCGATTTGT 538

UCSC_Genome CGGGAAAACTTGCTTTTTTACTTGCGTTTTATGTTCAACACACTAAGTTAATCGATTTGT 540

************************************************************

Our_Genome GTCTGGACAACTTGAACGTATGGTAGTTTTGTTTACAGTGTATCGAGTACATTTCATTTT 598

UCSC_Genome GTCTGAACAACTTGAATGTATGGTAGTTTTGTTTACAGAGTAGCGAGTACATTTCATTTT 600

***** ********** ********************* *** *****************

Our_Genome CGGGTGAACTGTCCCTTTAAGAGTGTGTTTGAAATGTCTCCGAGCAGCTTTTAATGCGAC 658

UCSC_Genome CGGGTGAACTGTCCCTTTAAGAGTGTGTTTAAAATGTCTCCGAGCAGCTTTTAATGCGAC 660

****************************** *****************************

Our_Genome CACAGCTGTACTAGTCACGAAAAGGGACCCGAAATCCATTTCAGCTTCTTTAAATAGCCA 718

UCSC_Genome CACAGCTTTACTAGTCACGAAAAGGGACCCGAAATCCATTTCAGCTTCTTTAAATAGCCA 720

******* ****************************************************

Our_Genome AGGGAAACTTTCATTGTGGTGAATATATCATAAATGTAGCTCGTATATCTCAGATGTAGC 778

UCSC_Genome AGGGAAATTTTCATTGTGGTGAATATATCATAAATGTAGCTCGTA--TCTCAGATGTAGC 778

******* ************************************* *************

Our_Genome TGTGATTCGGATTGTATGGAAGCTCATATGAGGACGGAACCAAAACAGCACTTGATTATG 838

UCSC_Genome TGTGATTCGGATTGTATGGAAGCTCATATGAGGACGGAACCAAAACAGCACTTGATTATG 838

************************************************************

Our_Genome TTAGCCTTATTATTTTGTAGTATTTATTTATTATTGGTTTAAAGCCATGTGAAGAGGAGG 898

UCSC_Genome TTAGCCTTATTATTTTGTAGTATTTATTTATTATTGGTTTAAAGCCATGTGAAGAGGAGG 898

************************************************************

Our_Genome CGGCCATATACAATGCATCTGTGATGATGCATTACAGCAGTCATCATTCTTTCCCCCATT 958

UCSC_Genome CGGCCATATACAATGCATCTGTGATAATGCATTACAGCAGTCATCATTCTTTCCCCCATT 958

************************* **********************************

Our_Genome AATGGATAATACATTAATAATGAAAGCATTAATGCTGGTTGTGAGATTGCGAATGAAGGC 1018

UCSC_Genome AATGGATGATACATTAATAATGAAAGCATTAATGCTGGTTGTGAGATTGTGAATGAAGGC 1018

******* ***************************************** **********

Our_Genome AATGTAATACATCACATGATGATATCATATGGTTTGTGGAGACAATCTGTTGCTGAGACT 1078

UCSC_Genome AATGTAATATATCACATGATGACATTATATGGTTTGTGGAGACAATCTATTGCTGAGACT 1078

********* ************ ** ********************** ***********

Our_Genome TGAGCGATTTACGTTAAGCTAATAAACCACACTACACAATTTATGATTTATTATAATCAT 1138

UCSC_Genome GGAGCGATTTACGTTAAGCTAATAAACCACACTACACAATTTATGATTTATTATAATCAT 1138

***********************************************************

Our_Genome GTTATTTATGATGTCCGCAGCGAGCCAGTGATTTATT------TTTTTTATTATTAATTA 1192

UCSC_Genome GTTATTTATGATGTCCGCAGCGAGCCTTTTTTTTATTATTATTTTTTTTATTATTAATTA 1198

************************** * ****** *****************

Our_Genome ACTTCATTAAGTTGAGCACAAACATTATAACTGGAGCATAAGCCATAGATTAAAAGCTAT 1252

UCSC_Genome ACTTCATGAAGTTGAGCACAAACATTATAACTGGAGCATAAGCCATAGATTAAAAGCTAT 1258

******* ****************************************************

Our_Genome GATTATGATTATTTTGGTTACTGAAATGACCCTTCACTGTTACATTTTTGTGATTTGTGG 1312

UCSC_Genome GATTATGATTATTTTGGTTACTGAAATGACCCTTCACTGTTACATTTTTGTGATTTGTGG 1318

************************************************************

Our_Genome GAATGTAATTATTATTATGATTTATAATCGAATGCTGCATGCCACGTATGTTTTTAATAA 1372

UCSC_Genome GAATGTAATTATTATTATGATTTATAATCGAATGCTGCATGCCACGTATGTTTTTAATAA 1378

************************************************************

Our_Genome AGCTGCCATTTTGTATGAT 1391

UCSC_Genome AGCTGCCATTTTGTATGAT 1397

*******************

cDNA CGAGACAAAACCACCCGCAGAATGAGAGAAACGCTGCTTAATTAAACACCCTCTCCATAC 60

Our_Genome CGAGACAAAACCACCCGCAGAATGAGAGAAACGCTGCTTAATTAAACACCCTCTCCATAC 60

************************************************************

cDNA AAAAACATCAGAATAAATCCGCCATCGACAACTCCGTCAAACTAAACAGCATCAGGTGAA 120

Our_Genome AAAAACATCAGAATAAATCCGCCATCGACAACTCCGTCAAACTAAACAGCATCAGGTGAA 120

************************************************************

cDNA TTTGTGCACAAAAAAAAACAAGTCCAGCGTGTAGCTGAGCGCGGAAATGCTTTAAAAAGG 180

Our_Genome TTTGTGCACAAAAAAAAACAAGTCCAGCGTGTAGCTGAGCGCGGAAATGCTTTAAAAAGG 180

************************************************************

cDNA GGATGGTTCACTCAAAAATGAAAACTACGGCATTAATTATTAGTCACAAACCTGTTAAAC 240

Our_Genome GGATGGTTCACTCAAAAATGAAAACTACGGCATTAATTATTAGTCACAAACCTGTTAAAC 240

************************************************************

cDNA TGACTTTCTTCAGTTGAATAAGAGAAGATATTTGGAAGAATGCTGAAAACCTGTAACCAT 300

Our_Genome TGACTTTCTTCAGTTGAATAAGAGAAGATATTTGGAAGAATGCTGAAAACCTGTAACCAT 300

************************************************************

cDNA TGACTTCCATGTTGTTTTTCATACTATGGAAGTCAATAGTTACAGCATTTTTCAAAATCT 360

Our_Genome TGACTTCCATGTTGTTTTTCATACTATGGAAGTCAATAGTTACAGCATTTTTCAAAATCT 360

************************************************************

cDNA CCTTTCGGGTTCAACAGAAAACAGAAACTCTTAACAGGTTTGGAAGCACTTGAGGGAGAC 420

Our_Genome CCTTTCGGGTTCAACAGAAAACAGAAACTCTTAACAGGTTTGGAAGCACTTGAGGGAGAC 420

************************************************************

cDNA TAACTAGACTAATACAATTACTAGCTCATAAAAAATGTGCTGAAACAACTGAAATTCTCG 480

Our_Genome TAACTAGACTAATACAATTACTAGCTCATAAAAAATGTGCTGAAACAACTGAAATTCTCG 480

************************************************************

cDNA GGAAAACTTGCTTTTTTACTTGCGTTTTATGTTCAACACACTAAGTTAATCGATTTGTGT 540

Our_Genome GGAAAACTTGCTTTTTTACTTGCGTTTTATGTTCAACACACTAAGTTAATCGATTTGTGT 540

************************************************************

cDNA CTGGACAACTTGAACGTATGGTAGTTTTGTTTACAGTGTATCGAGTACATTTCATTTTCG 600

Our_Genome CTGGACAACTTGAACGTATGGTAGTTTTGTTTACAGTGTATCGAGTACATTTCATTTTCG 600

************************************************************

cDNA GGTGAACTGTCCCTTTAAGAGTGTGTTTGAAATGTCTCCGAGCAGCTTTTAATGCGACCA 660

Our_Genome GGTGAACTGTCCCTTTAAGAGTGTGTTTGAAATGTCTCCGAGCAGCTTTTAATGCGACCA 660

************************************************************

cDNA CAGCTGTACTAGTCACGAAAAGGGACCCGAAATCCATTTCAGCTTCTTTAAATAGCCAAG 720

Our_Genome CAGCTGTACTAGTCACGAAAAGGGACCCGAAATCCATTTCAGCTTCTTTAAATAGCCAAG 720

************************************************************

cDNA GGAAACTTTCATTGTGGTGAATATATCATAAATGTAGCTCGTATATCTCAGATGTAGCTG 780

Our_Genome GGAAACTTTCATTGTGGTGAATATATCATAAATGTAGCTCGTATATCTCAGATGTAGCTG 780

************************************************************

cDNA TGATTCGGATTGTATGGAAGCTCATATGAGGACGGAACCAAAACAGCACTTGATTATGTT 840

Our_Genome TGATTCGGATTGTATGGAAGCTCATATGAGGACGGAACCAAAACAGCACTTGATTATGTT 840

************************************************************

cDNA AGCCTTATTATTTTGTAGTATTTATTTATTATTGGTTTAAAGCCATGTGAAGAGGAGGCG 900

Our_Genome AGCCTTATTATTTTGTAGTATTTATTTATTATTGGTTTAAAGCCATGTGAAGAGGAGGCG 900

************************************************************

cDNA GCCATATACAATGCATCTGTGATGATGCATTACAGCAGTCATCATTCTTTCCCCCATTAA 960

Our_Genome GCCATATACAATGCATCTGTGATGATGCATTACAGCAGTCATCATTCTTTCCCCCATTAA 960

************************************************************

cDNA TGGATAATACATTAATAATGAAAGCATTAATGCTGGTTGTGAGATTGCGAATGAAGGCAA 1020

Our_Genome TGGATAATACATTAATAATGAAAGCATTAATGCTGGTTGTGAGATTGCGAATGAAGGCAA 1020

************************************************************

cDNA TGTAATACATCACATGATGATATCATATGGTTTGTGGAGACAATCTGTTGCTGAGACTTG 1080

Our_Genome TGTAATACATCACATGATGATATCATATGGTTTGTGGAGACAATCTGTTGCTGAGACTTG 1080

************************************************************

cDNA AGCGATTTACGTTAAGCTAATAAACCACACTACACAATTTATGATTTATTATAATCATGT 1140

Our_Genome AGCGATTTACGTTAAGCTAATAAACCACACTACACAATTTATGATTTATTATAATCATGT 1140

************************************************************

cDNA TATTTATGATGTCCGCAGCGAGCCAGTGATTTATTTTTTTTATTATTAATTAACTTCATT 1200

Our_Genome TATTTATGATGTCCGCAGCGAGCCAGTGATTTATTTTTTTTATTATTAATTAACTTCATT 1200

************************************************************

cDNA AAGTTGAGCACAAACATTATAACTGGAGCATAAGCCATAGATTAAAAGCTATGATTATGA 1260

Our_Genome AAGTTGAGCACAAACATTATAACTGGAGCATAAGCCATAGATTAAAAGCTATGATTATGA 1260

************************************************************

cDNA TTATTTTGGTTACTGAAATGACCCTTCACTGTTACATTTTTGTGATTTGTGGGAATGTAA 1320

Our_Genome TTATTTTGGTTACTGAAATGACCCTTCACTGTTACATTTTTGTGATTTGTGGGAATGTAA 1320

************************************************************

cDNA TTATTATTATGATTTATAATCGAATGCTGCATGCCACGTATGTTTTTAATAAAGCTGCCA 1380

Our_Genome TTATTATTATGATTTATAATCGAATGCTGCATGCCACGTATGTTTTTAATAAAGCTGCCA 1380

************************************************************

cDNA TTTTGTATGAT 1391

Our_Genome TTTTGTATGAT 1391

***********
