# Supplementary material for: Deletion of the WD40 Domain of LRRK2 in Zebrafish Causes Parkinsonism-Like Loss of Neurons and Locomotive Defect
Source: PLoS Genet. 2010 Apr 22;6(4):e1000914. doi: 10.1371/journal.pgen.1000914 (PMC2858694; doi:10.1371/journal.pgen.1000914)
Supplement: Protocol S1 — Supporting materials and methods. (0.04 MB DOC) [file pgen.1000914.s012.doc]

**Supporting materials and methods**

**Zebrafish (Danio rerio) maintenance**

Embryos were maintained in egg water [1 L Milli Q water supplemented with 1.5 ml stock salts, The Zebrafish Book (Westerfield, 1995)] and placed in an incubator at 28.5ºC. Embryos older than 24 hours post fertilization (hpf) were raised in 0.003% 1-phenyl-2-thiourea (PTU, Sigma) in egg water to inhibit the production of pigment. The embryos were constantly monitored under the microscope for staging. Time of development at 28.5ºC and morphological features (Kimmel et al., 1995) were used to stage.

**Northern Analysis**

PCR product of zLRRK2 was cloned into pCR-Blunt II TOPO (Invitrogen) for probe synthesis. Northern probe is labeled by DIG using Roche’s DIG labeling kit. 10ug RNA from different tissues was used for agarose gel separation and transfer to the membrane (Ambion). After fixation and blocking using blocking buffer from Ambion, the probes (1ul) were used for overnight hybridization in hybridization buffer from ambion as well at 68ºC. After washing, the antibody labeled by AP was subjected to detection of DIG and film development.

**Western analysis**

We attempted to develop anti-zLRRK2 antibodies using two synthesized peptides from the N-terminal (CHLAEFIEKKDHEVV) and the C-terminal (within WD40 domain, CSTRKPKVHSEDQSR) regions of LRRK2. After affinity purification using the peptides, the specificity of the purified antibodies was determined by Western blot analyses. Using the antibody recognizing the C-terminal region of zLRRK2, a protein with a molecular weight of 280kD could be detected specifically from adult brain homogenate, but not from other tissues (Figure S10). Furthermore, this specific signal of the Western blot from brain homogenate and zLRRK2-overexpressing Cos-7 cells could be blocked by the peptide (1:100) pre-incubated with the antibody for C-terminal region (Figure S10), suggesting that this antibody could recognize the *zLRRK2* protein. No specific proteins could be detected by the antibody against the N-terminal region (data not shown). 60µg total proteins tissues (brain, muscle, gut and ovary) and 40μg total protein from LRRK2-overexpressing cell lines were extracted and used for SDS-PAGE and Western blot. Briefly, proteins were separated by 4-12% gradient gel, and they were transferred onto PVDF membrane (soaked with methanol for 1-5 min before use) at 20V for overnight at 4ºC. The membrane was blocked in 5% nonfat dry milk for 1 hour. It was then incubated with diluted primary antibodies (1: 1000 for anti-zLRRK2 and anti-hLRRK2 antibodies (NOVUS NB 300-268); 1:5000 for anti-TH and 1:10000 for anti-β-actin and GAPDH antibodies) in 5% nonfat dry milk for at least for 2 hours at room temperature. After 3 times stringent TBS-T washes, the membrane was incubated with diluted secondary antibodies (1:5000 for HRP-conjugated anti-rabbit or anti-mouse IgG) for 1 hour. Signals were visualized by developing the membrane with ECL Plus Western Blotting Detection Kit (GE Healthcare) after 3 stringent TBS-T washes.

**Acetylated-tubulin staining on the brain**

Embryos at 3 dpf were fixed in 2% TCA in PBST (phosphate-buffered saline + 1% Triton X-100) for several hours at room temperature or overnight at 4°C. After several washes in PBST, the embryos were treated with 1 mg/ml collagenase (Sigma) in PBS for 10–20 minutes on a nutator. After 20 minutes or if they started to fall apart, embryos were washed several times with PBST to stop collagenase digestion. They were then transferred to a Petri dish and gently swirled in PBST. During this process, the embryos would fall apart, leaving individual eyes and brains intact. Brains often remained attached to the spinal cord. Isolated brains were washed in blocking solution (5% normal goat serum/2% BSA in PBST) for one hour. Anti-acetylated tubulin (Sigma) (1:1000) incubation was done in blocking solution at 4°C overnight on a nutator, followed by several washes in PBST. An Alexa568 anti-mouse secondary antibody (1:500) was done overnight at 4°C or several hours at room temperature.

**Phylogenetic Analysis**

All protein sequences are aligned using ClustalX version 1.83 (Thompson et al. 1997). By the Neighbor-joining (NJ) algorithm (Saitou and Nei 1987) in ClustalX 1.83, a preliminary phylogenetic trees for both COR and Kinase domains are obtained. Based on the trees, similar/duplicate sequences from the same organism are removed. Then, 26 and 18 animal sequences are obtained for the COR and Kinase domains, respectively.

Those sequences for the COR and Kinase domains are aligned again by ClustalX 1.83. Using the Neighbor-joining (NJ) algorithm implemented in MEGA 3.1 (Kumar, Tamura, and Nei 2004) and the Maximum Parsimony (MP) algorithm implemented in PAUP* beta 10 version (Swofford 2003), the phylogenetic trees for the COR and Kinase domains are reconstructed by performing the bootstrap analyses with 1000 replicates. The trees reconstructed by both methods are the same and they are shown in Figure S1.

**Splicing interfering mopholino design**

Since first exon deletion within WD40 domain won’t cause any reading frame shift, second exon within WD40 domain was targeted which is the 45^th^ exon in zlrrk2 cDNA sequence shown as following (UPPERCASE is 45^th^ exon):

tgctagaaat gtgcatcatt tagccagcta tttactataa cagtgtactg

ttcctaatgc tgtgtttcag GAGCTGGACC GCAGCCCTAT CCTCTGCATG

GTGATCATCA GAGCTGCAGA CTCGTGCAGT GACTGGCTAG TGGCTGGTTC

AGAGTCCGGT TCGCTCTTTA TCATGGACAC CATTAATGCG AAAGTCCTGC

ACCGTCTGAA GAGTGTGAAG GACTCTGTGA CGTCTCTGTA CTTTCACACC

GAGCTCCAGC ACAGgttaat aaatctgctt gtgacacacg cttggttgat

ctagcttaaa ttgaaaaaat gatgcttatt tttgtctgca

Basing the sequence information above (intron: lower case; exon: upper case), two splicing morpholinos were designed to cause the deletion of 45^th^ exon: intron 45 splice donor junction; EI – CACAAGCAGATTTATTAACCTGTGC (targeted position highlighted in blue); intron 44 splice acceptor junction, IE – GCTCCTGAAACACAGCATTAGGAAC (targeted position highlighted in red). Either of the morpholinos alone can cause the deletion of 45^th^ exon.

RT-PCR and sequencing result of WD40 morphant showed deletion of second exon within WD40 domain which will cause reading frame shift and introduce a premature stop codon which is shown as following (highlighted in red color):

1 AACTCTGCAGAGATGTTGTGTCTGACGCGAGAGCTGAATGTTGTGGGTTTTCCAGGCGAG

1 N S A E M L C L T R E L N V V G F P G E

61 TGTTTTGTCGTGTCCAATTCAGGCGGAGCTGCAAACGGAGGTAAAAACCCTCATGTGTGG

21 C F V V S N S G G A A N G G K N P H V W

121 ATCGGTGGTGGCAGCAGCAGCCAGAAACTGGGCTGTGTGACTGCGGTGGATCTGGAGACC

41 I G G G S S S Q K L G C V T A V D L E T

181 GGCGGGAGTTTGAACCAGATGTCTGAAGAGTTATCTTTTGGTGGGCACAGCAGACGGGAC

61 G G S L N Q M S E E L S F G G H S R R D

241 ACTGGTCATTTATGAAGATTCAGCCCTGAAGCTGGAGAACGGTGGTCCGGTGAAGACGCT

81 T G H L *

Alignment of amino acid sequence between WD40 domain of WT and WD40 morphant (Critical amino acid N in WD40 domain is highlighted in red):

WD40_WT NSAEMLCLTRELNVVGFPGECFVVSNSGGAANGGKNPHVWIGGGSSSQKLGCVTAVDLET 60

WD40_MO NSAEMLCLTRELNVVGFPGECFVVSNSGGAANGGKNPHVWIGGGSSSQKLGCVTAVDLET 60

************************************************************

WD40_WT GGSLNQELDRSPILCMVIIRAADSCSDWLVAGSESGSLSIMDTINAKVLHRLKSVKDSVT 120

WD40_MO GGSLNQMSE--------------------------------------------------- 69

****** :

WD40_WT SLYFHTELQHRCLKSYLLVGTADGTLVIYEDSALKLENGGPVKTLEVGDVNTPLMCLGPS 180

WD40_MO ----------------------------------ELSFGG-------------------- 75

:*. **

WD40_WT SHPQERRSLWAACGTRIILFTVEFDVCRSIDTKPKPLFPLQARVSGEACISILAVDKHVY 240

WD40_MO ---HSRR-----------------------DT---------------------------- 81

:.** **

WD40_WT VSKTGGHTVEVWDKKTERMVNLIDCMQLLGLSSTRKPKVHSEDQSRPMVPSLVVKALLVQ 300

WD40_MO -----GHL---------------------------------------------------- 84

**

WD40_WT HSGTLWIGTRAGHILLVEVSSCHLLQTINPHCHSIRCMSSILLDTLNRKNVILVLGRRQR 360

WD40_MO ------------------------------------------------------------

WD40_WT IHLEQLKTQSGEDSVLTLWDKKTERMVNLIDCMQLL 396

WD40_MO ------------------------------------
